# MdNup62 interactions with MdHSFs involved in flowering and heat-stress tolerance in apple

**DOI:** 10.1186/s12870-022-03698-3

**Published:** 2022-07-04

**Authors:** Chenguang Zhang, Na An, Peng Jia, Wei Zhang, Jiayan Liang, Hua Zhou, Dong Zhang, Juanjuan Ma, Caiping Zhao, Mingyu Han, Xiaolin Ren, Libo Xing

**Affiliations:** College of Horticulture, Northwest A&F University, 3 Taicheng Road, Yangling, 712100 Shaanxi, China

**Keywords:** Apple, Flowering, Heat stress, Nuclear pore complex, *MdNup62*, *MdHSFs*

## Abstract

Because of global warming, the apple flowering period is occurring significantly earlier, increasing the probability and degree of freezing injury. Moreover, extreme hot weather has also seriously affected the development of apple industry. Nuclear pore complexes (NPCs) are main channels controlling nucleocytoplasmic transport, but their roles in regulating plant development and stress responses are still unknown. Here, we analysed the components of the apple NPC and found that MdNup62 interacts with MdNup54, forming the central NPC channel. MdNup62 was localized to the nuclear pore, and its expression was significantly up-regulated in ‘Nagafu No. 2’ tissue-cultured seedlings subjected to heat treatments. To determine *MdNup62*’s function, we obtained *MdNup62*-overexpressed (OE) Arabidopsis and tomato lines that showed significantly reduced high-temperature resistance. Additionally, OE-*MdNup62* Arabidopsis lines showed significantly earlier flowering compared with wild-type. Furthermore, we identified 62 putative MdNup62-interacting proteins and confirmed MdNup62 interactions with multiple MdHSFs. The OE-*MdHSFA1d* and OE-*MdHSFA9b* Arabidopsis lines also showed significantly earlier flowering phenotypes than wild-type, but had enhanced high-temperature resistance levels. Thus, MdNUP62 interacts with multiple MdHSFs during nucleocytoplasmic transport to regulate flowering and heat resistance in apple. The data provide a new theoretical reference for managing the impact of global warming on the apple industry.

**Supplementary Information:**

The online version contains supplementary material available at 10.1186/s12870-022-03698-3.

## Introduction

Apple (*Malus* × *domestica* Borkh.) is a widely cultivated and economically important fruit crop in temperate regions worldwide owing to its high nutritional value, good storage, and lengthy supply period. And Fuji apple is the main cultivar in China, but there are cultivation and production problems, including flowering difficulties and severe alternate bearing [1, 2]. However, with global warming, an increase in the average temperature in winter will result in earlier apple flowering [3, 4], and if there is cold weather in early spring, then significant flower and fruit losses will result. Additionally, at present, extreme hot weather occurs frequently in summer, causing other problems, such as growth impairment and production decline [5, 6], which have seriously affected the development of the apple industry in China.

Floral induction pathways have been extensively studied, and there are six signalling pathways in the model plant *Arabidopsis thaliana*, including photoperiodic, vernalization, autonomic, gibberellin, temperature-sensitive, and age pathways[7–9]. In apple, the functions of some key flowering-related genes have been well studied in recent years, such as *APETALA1* (*AP1*), *LEAFY (LFY*), *FLOWERING LOCUS T* (*FT)*, and *TERMINAL FLOWER 1*(*TFL1*). For instance, overexpression of *MdMADS5*, a putative homolog of *AP1*, leads to significant early flowering in Arabidopsis [10]. Apple anti-*TERMINAL FLOWER 1* transgenic lines flower significantly earlier than the WT, with the earliest flowering at 8 months, while the WT did not flower for 6 years [11]. Through transcriptome analyses, the induction of apple flower buds was found to be regulated by sugar and hormone signalling pathways [12]. Other omics studies have revealed the molecular mechanisms involved in responses to exogenous treatments, such as sugar [13], 6-benzylaminopurine [14], and gibberellins [15], and their effects on the flowering of apples. However, research on apple flowering is still relatively limited.

A nuclear pore complex (NPC) is composed of a class of nucleoporins (Nups) located in the nuclear pore [16]. More than 30 *Nups* have been identified in Arabidopsis and 38 members have been identified in apple [16, 17]. Some Nups interact and form three subcomplexes: Nup62, Nup93, and Nup107–160 [16, 18]. *Nups* control the transport of substances, such as RNA and proteins, between the nucleus and cytoplasm [19, 20], and play important roles in regulating plant growth and development, as well as biotic and abiotic stresses [19, 21, 22]. For example, *HOS1*, *Nup96*, *Nup54*, *Nup58*, *Nup62*, *Nup136*, and *Nup160* are important for plant flowering [16, 23–26]. *HOS1*, *Nup85*, *Nup96*, and *Nup133* participate in abiotic stress pathways [18, 20, 27–29]. *MOS7*, *Nup96*, *Nup160*, and *Sec1* play important roles in plant immunity [30–32], and *Nup96*, *Nup160*, and *TPR* affect hormone signalling pathways [33–37].

Heat shock factors (*HSFs*) are important components of signal transduction and play important roles in diverse stress pathways [38]. The *HSF* family in plants has more members (21 *HSFs* in *Arabidopsis*) and more complex regulatory mechanisms [39, 40] than in vertebrates (4 *HSFs*) or Drosophila (only 1 *HSF*). On the basis of their structural differences, HSFs may be divided into three classes, A, B, and C [39]. Class A has the C-terminal short peptide AHA domain, which has an activator function, while the B and C classes lack this domain [41]. HSFs specifically identify and bind heat shock elements (HSEs), which contain nGAAnnTTCn or nTTCnnGAAn in the downstream target genes’ promoters [42]. Class A members (*HSFA1a*, *HSFA1b*, *HSFA1d*, *HSFA1e*, *HSFA2*, and *HSFA3*) positively regulate plant heat tolerance [43–47], while, in contrast, Class B *HSFs* (*HSFB1* and *HSFB2b*) negatively regulate heat-induced *HSFs* and plant heat tolerance [48]. In addition to responding to heat stress, some *HSFs* (*HSFA2*, *HSFA1E*, and *HSFA4C*) appear to be involved in plant flowering pathways [49, 50].

Currently, there are no reported functional studies of Nups in apple. *Nup62* is a member of the Nup62 subcomplex in the central core of the nuclear pore [16, 17], and *nup62 A. thaliana* mutants have been reported to flower early, indicating *Nup62*’s involvement in flowering pathways [25]. In this study, we characterized apple *Nup62*, which showed a high transcription level at the flower bud developmental stage and was responed to high temperature. The overexpression of *MdNup62* in Arabidopsis resulted in earlier flowering compared with WT. Moreover, The overexpression of *MdNup62* in Arabidopsis and tomato both reduced heat resistance. Further, we performed a yeast two-hybrid (Y2H) sieve library experiment to screen for proteins that interact with MdNup62, and the interactions between MdNup62 and the MdHSFs were confirmed. And the overexpression of *MdHSFA1d* and *MdHSFA9b* independently in Arabidopsis resulted in earlier flowering and enhancing heat resistance. Thus, *MdNup62* and the *MdHSFs* regulate flowering and respond to temperature changes. These results provide a theoretical reference for managing the impact of global warming on the apple industry.

## Results

### Apple NPC structure and composition, and its expression patterns

**Fig. 1:**
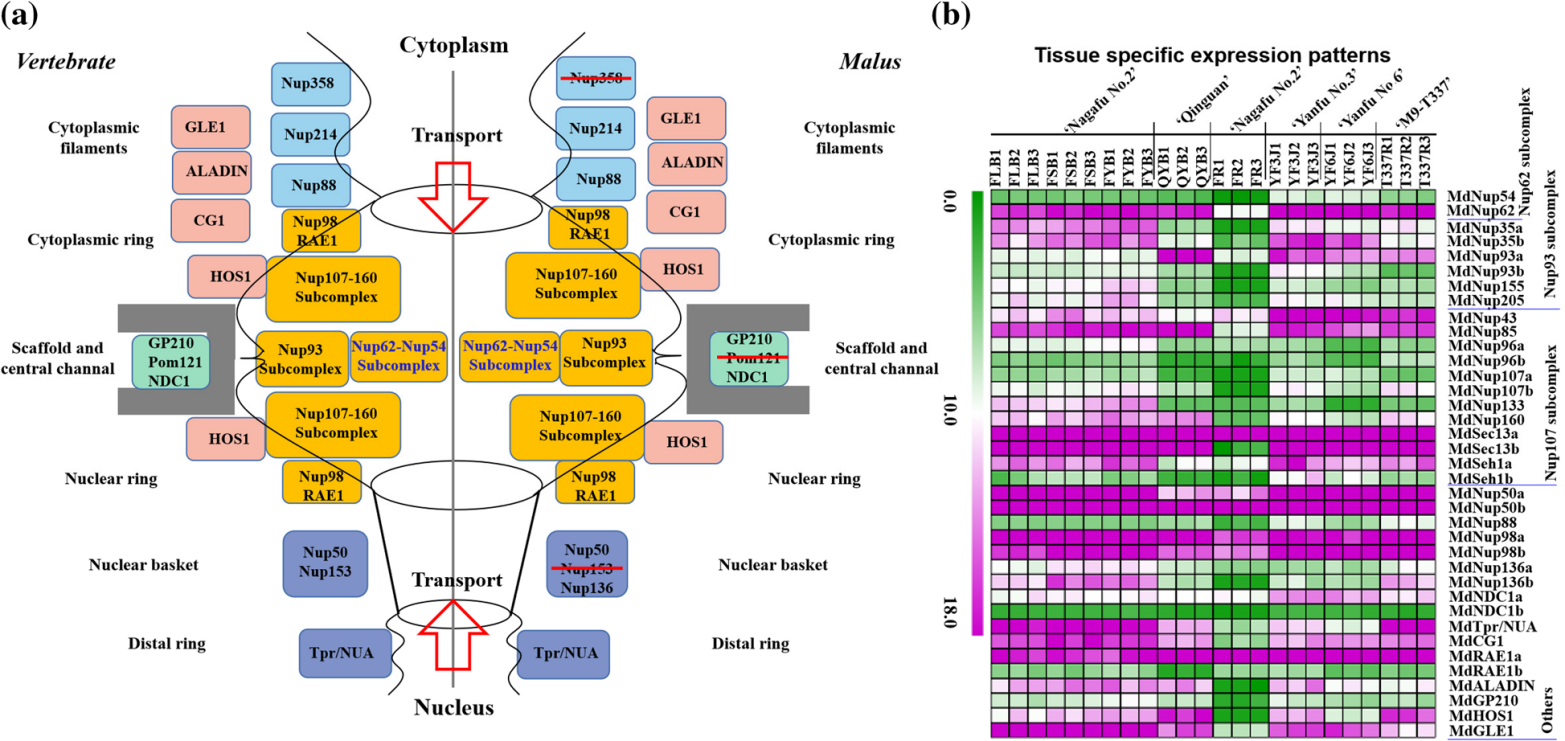
The nuclear pore complex (NPC) structure and composition in *Vertebrate* and *Malus.* **a** A schematic of the nuclear pore with the cytoplasmic side at the top and the nuclear basket at the bottom for Vertebrate (left) and Malus (right). **b** Tissue specific expression patterns of apple NPC components by RNA-seq. The full names of the different abbreviations are as follows, ‘Nagafu No.2’ long branches flower buds (FLB), ‘Nagafu No.2’ short branches flower buds (FSB), ‘Nagafu No.2’ axillary buds (FYB), ‘Qinguan’ axillary buds (QYB), ‘Nagafu No.2’ fruit (FR), ‘Yanfu No.3’ stem tip (YF3J), ‘Yanfu No.6’ stem tip (YF6J), and ‘M9-T337’ root (T337R). Each number after the abbreviation represents a biological repetition

Compared with vertebrate, apple NPC consists of 38 Nup proteins, but missing some Nups, such as Nup153, Nup358, Pom121, etc. Refer to the structure of vertebrate NPC [16], we devided apple NPC into five parts: Cytoplasmic filaments (Nup214 and Nup88), Cytoplasmic and Nuclear ring (Nup98, RAE1, and Nup107-160 Subcompex), Scaffold and central channal (GP210, NDC1, Nup62 Subcompex, and Nup93 Subcompex), Nuclear basket (Nup50 and Nup136), and Distal ring (Tpr/NUA), as well as GLE1, ALADIN, CG1, and HOS1 also participate in NPC constitution (Fig. 1a). Additionally, MdNup62 interacts with MdNup54 on Y2H and LUC experiment, and they might forming the central apple NPC channel involving in nucleocytoplasmic transport (Fig. S1) [17].

We examined the expression patterns of NPC components in different tissues of several apple varieties (Fig. 1b; Table S1). The expression levels of *MdNup62* as central channel component showed significantly higher in buds, stem, roots than in fruit of apples, but other channel component *MdNup54* showed significantly low expression levels in all tissues compared with *MdNup62*, indicating that *MdNup62* play a key role in regulation of growth and stress response by controlling nucleocytoplasmic transport in apple.

### Feature, expression, and subcellular localization analyses of *MdNup62*

**Fig. 2:**
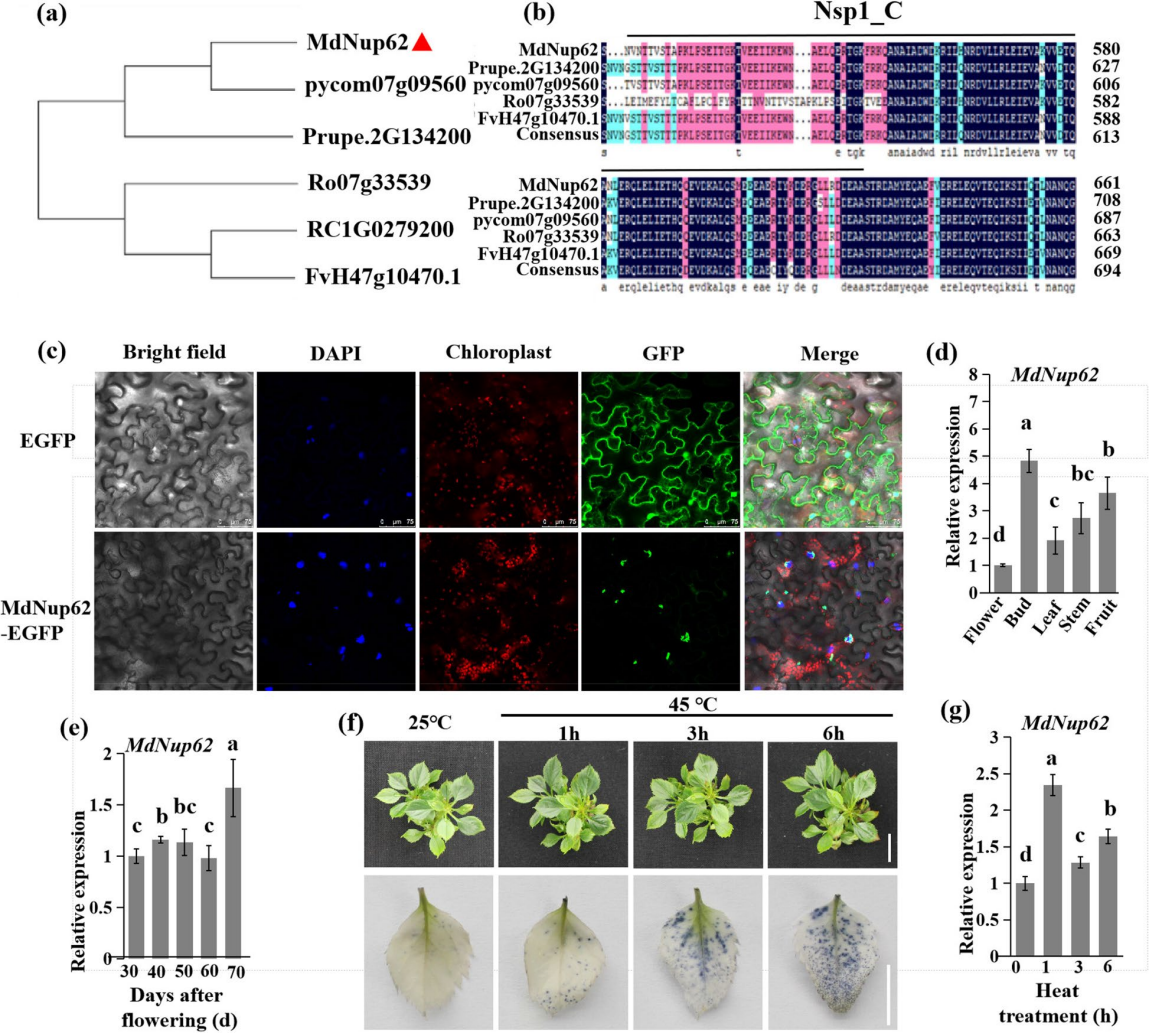
Identification and analysis of MdNup62. **a** Phylogenetic analysis of Rosaceae *Nup62*. **b** The conservative domain of Rosaceae Nup62. **c** Subcellular localization of MdNup62. The upper panel shows 35S::EGFP, and the lower panel shows 35S::MdNup62-EGFP. **d** and **e** Analyses of *MdNup62* expression levels in diverse ‘Nagafu No. 2’ apple tissues **d** and in different flower bud developmental stages of ‘Nagafu No. 2’ **e**. (f) The phenotype of ‘Nagafu No. 2’ tissue-cultured seedlings (upper panels) and the in situ accumulation of superoxide radical (O^2−^) at 0, 1, 3, and 6 h under heat treatment conditions (lower panels). Bar = 1 cm. **g** *MdNup62* expression levels in ‘Nagafu No. 2’ tissue-cultured seedling leaves at 0, 1, 3, and 6 h under heat-treatment conditions. Each sample was analysed with three biological replicates, each comprising three technical replicates. Means followed by different lowercase letters are significantly different at the 0.05 level. The same below

We initially performed a simple bioinformatics analysis of *MdNup62*. A phylogenetic tree of *Nup62* from six Rosaceae plants (*Rosa chinensis*, *Pyrus communis*, *Prunus persica*, *M. domestica*, *Rubus occidentalis*, and *Fragaria vesca*) was constructed using MEGA-X. *MdNup62* was most closely related to the *Nup62* of pear (Fig. 2a). The aligned protein sequences revealed a conserved Nsp1_C domain (Fig. 2b). The subcellular localization of MdNup62 was determined by introducing 35S::*MdNup62*-GFP into tobacco leaves (Fig. 2c). Tobacco leaves transformed with the empty vector 35S::GFP were used as controls. In the tobacco leaves expressing 35S::*MdNup62*-GFP, the GFP signal was observed only in the nuclear pore, while the GFP signal was detected throughout the control tobacco leaf cells, indicating that MdNup62 localized to the nuclear pore.

The transcript levels of *MdNup62* in different tissues were determined using qRT-PCR (Fig. 2d). The highest expression level was in flower buds. An *MdNup62* expression analysis during the flower bud developmental stages revealed that the expression level was stable at 30 to 60 days after flowering and reached its highest level at 70 days after flowering (Fig. 2e). Thus, *MdNup62* maintained a high expression level during flower bud induction, indicating that it may be related to bud differentiation in apple.

We exposed apple tissue-cultured seedlings to a heat treatment. The reactive oxygen species (ROS) accumulation in leaves increased from 0 to 6 h under heat-treatment conditions (Fig. 2f). Moreover, the expression level of *MdNup62* was determined at different times during the high-temperature treatment (Fig. 2g). *MdNup62* was significantly induced by high temperature, and its expression level was highest at 1 h after exposure to the high temperature. Thus, *MdNup62* may be involved in the heat-resistance pathway of apple.

### Overexpression of* MdNup62* promotes flowering

**Fig. 3:**
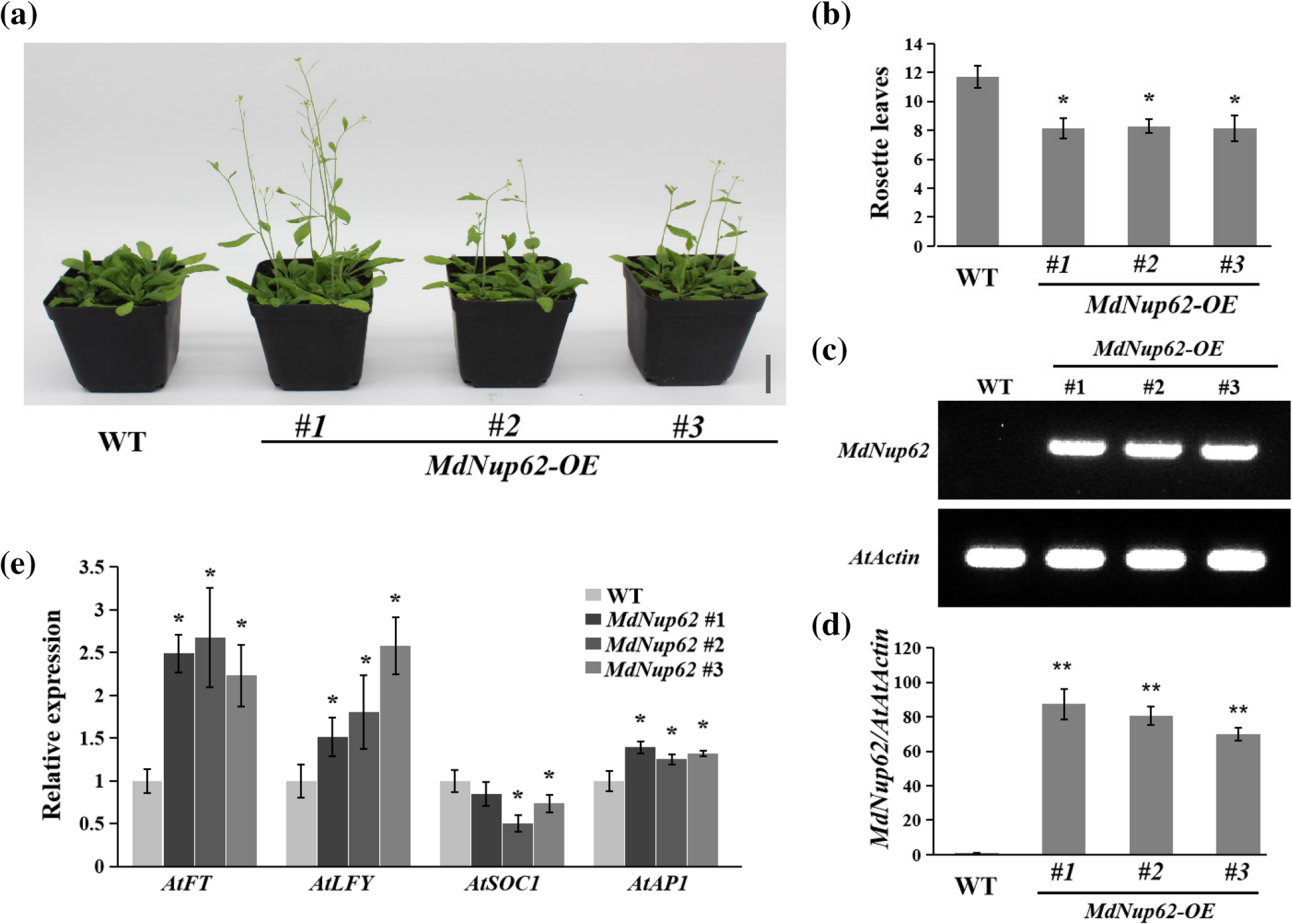
*MdNUP62* promotes flowering in Arabidopsis. **a** Phenotype of the *MdNUP62*-overexpression Arabidopsis line for flowering time. Bar = 2 cm. **b** Statistical analysis of rosette leaves of *Arabidopsis thaliana* during bolting. Asterisks denote significant differences as determined by a t-test (*P < 0.05).The same below. **c** Semi-quantitative RT-PCR analysis of *MdNup62* expression in Arabidopsis samples. **d** qRT-PCR analysis of *MdNup62* expression in Arabidopsis samples. **e** Relative expression levels of flowering genes (*AtFT*, *AtLFY*, *AtSOC1*, and *AtAP1*) in WT and *MdNup62*-overexpression lines

To confirm *MdNup62*'s role in flowering, we performed an Agrobacterium-mediated genetic transformation of *MdNup62* into *A. thaliana*. We found that OE-*MdNup62* lines flowered significantly earlier than WT (Fig. 3a). Additionally, OE-*MdNup62* lines had significantly fewer rosette leaves than WT during bolting (Fig. 3b). The presence of the transgene in OE-*MdNup62* lines was confirmed using genomic PCR (Figure S2a), semi-quantitative RT-PCR (Fig. 3c), and qRT-PCR (Fig. 3d). The transcript levels of flowering-related genes were analysed by qRT-PCR (Fig. 3e). The expression levels of *AtFT*, *AtLFY*, and *AtAP1* significantly increased in OE-*MdNup62* lines compared with WT. This demonstrated that the overexpression of *MdNup62* promoted flowering in Arabidopsis.

### Overexpression of *MdNup62* reduces high-temperature resistance

**Fig. 4:**
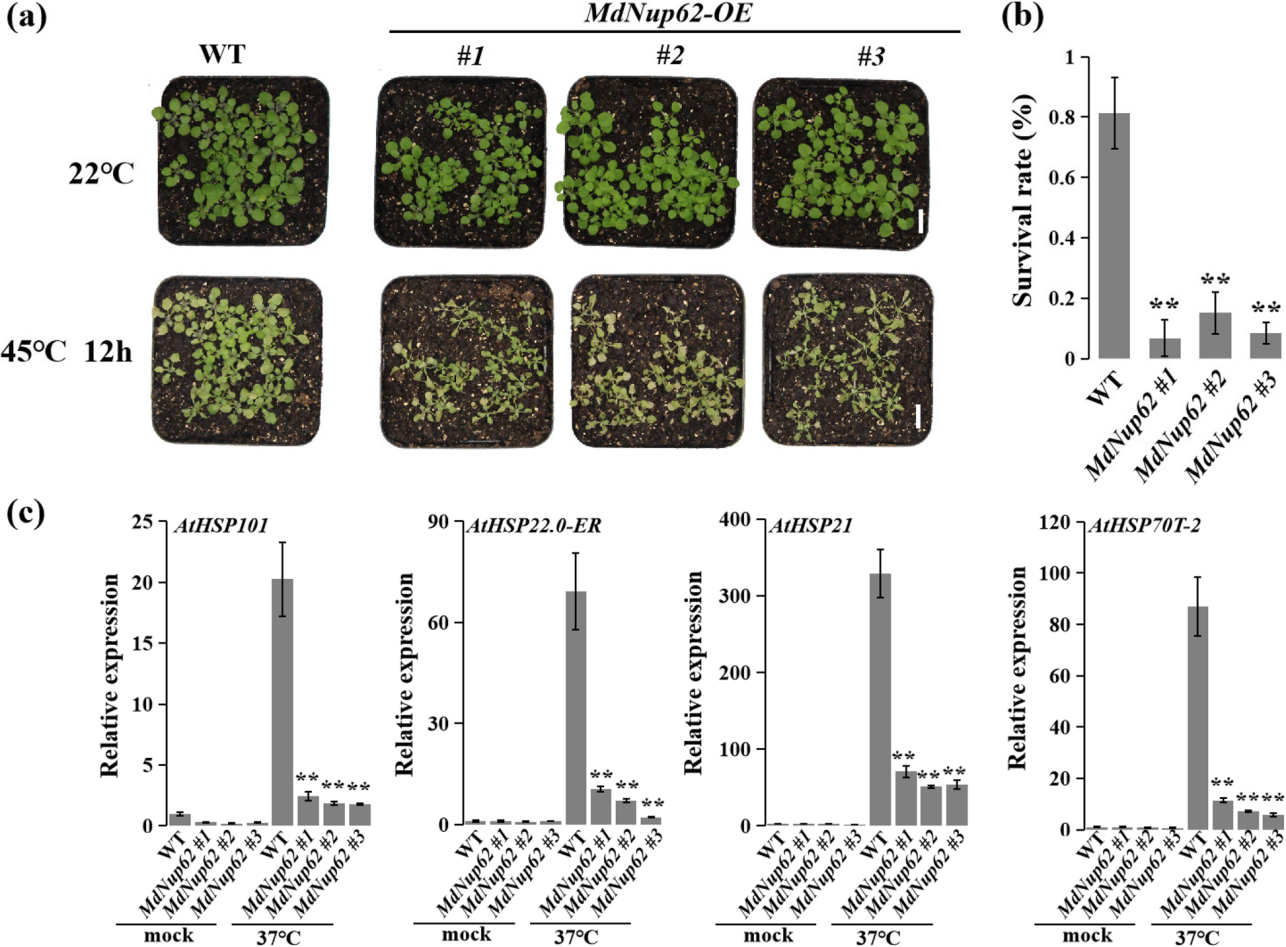
*MdNup62* reduced high-temperature resistance in Arabidopsis. **a** Phenotype of the *MdNup62*-overexpression Arabidopsis line for high-temperature resistance. Bar = 1 cm. **b** Survival rates of WT and *MdNup62*-overexpression Arabidopsis lines after the high-temperature treatment. **c** Relative expression levels of high-temperature resistance-related genes (*AtHSP101*, *AtHSP22.0-ER*, *AtHSP21*, and *AtHSP70T-2*) in WT and *MdNup62*-overexpression lines at the normal (22 °C) temperature and 1 h after exposure to the high-temperature (37 °C) treatment

**Fig. 5:**
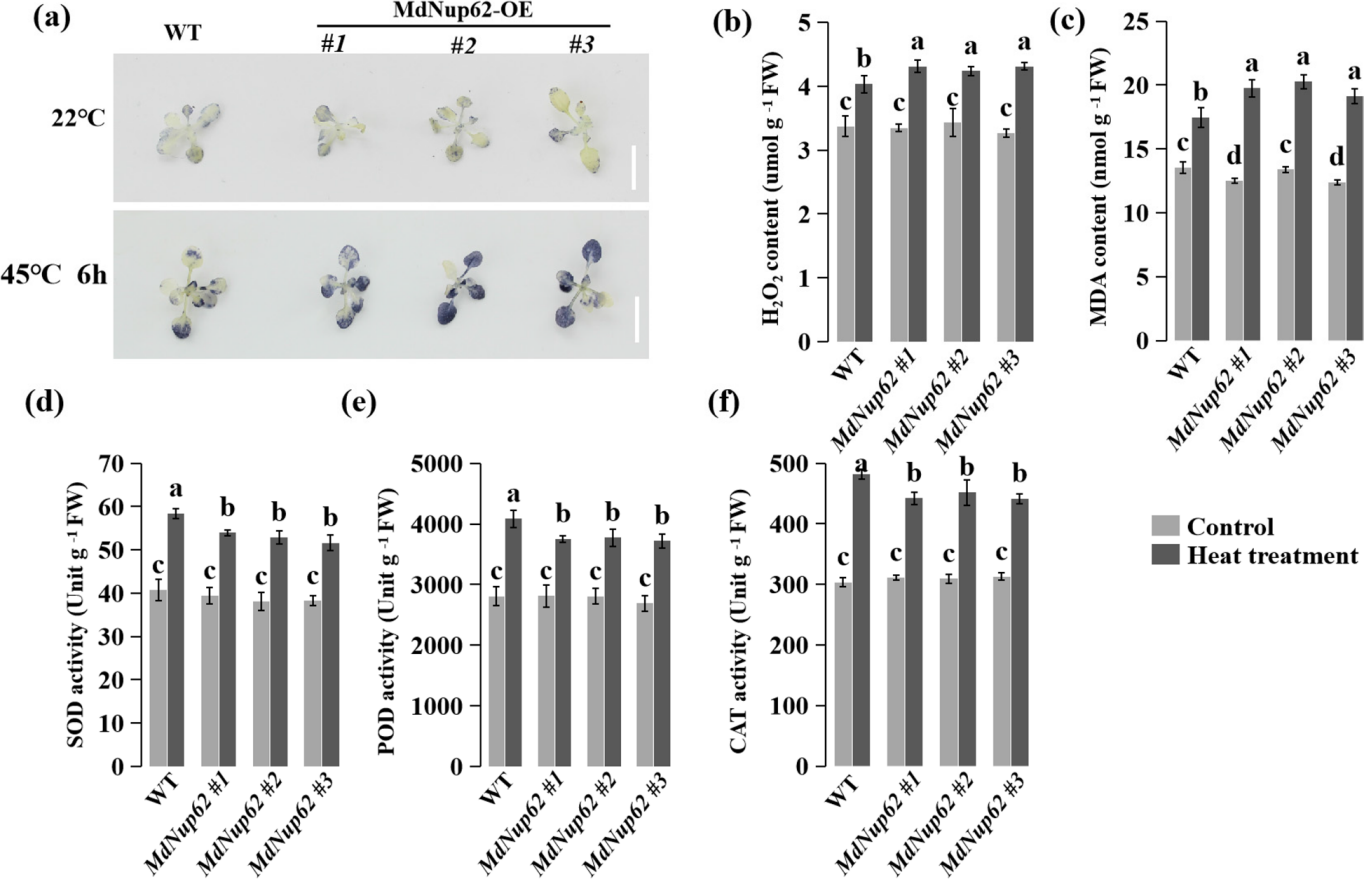
Changes in the level of accumulated ROS and activities of ROS-scavenging enzymes in *OE-MdNup62* and WT Arabidopsis leaves under heat-stress conditions. **a** In situ accumulations of superoxide radicals (O.^2−^) before (upper panels) and after (lower panels) heat treatment as revealed by nitro blue tetrazolium staining. Changes in the level of accumulated ROS **b** and **c** Quantitative measurement of H_2_O_2_c– and malondialdehyde concentrations in Arabidopsis leaves treated with and without the high temperature. **d**–**f** Activities of superoxide dismutase (SOD), peroxidase (POD), and catalase (CAT) at 6 h after the heat treatment

**Fig. 6:**
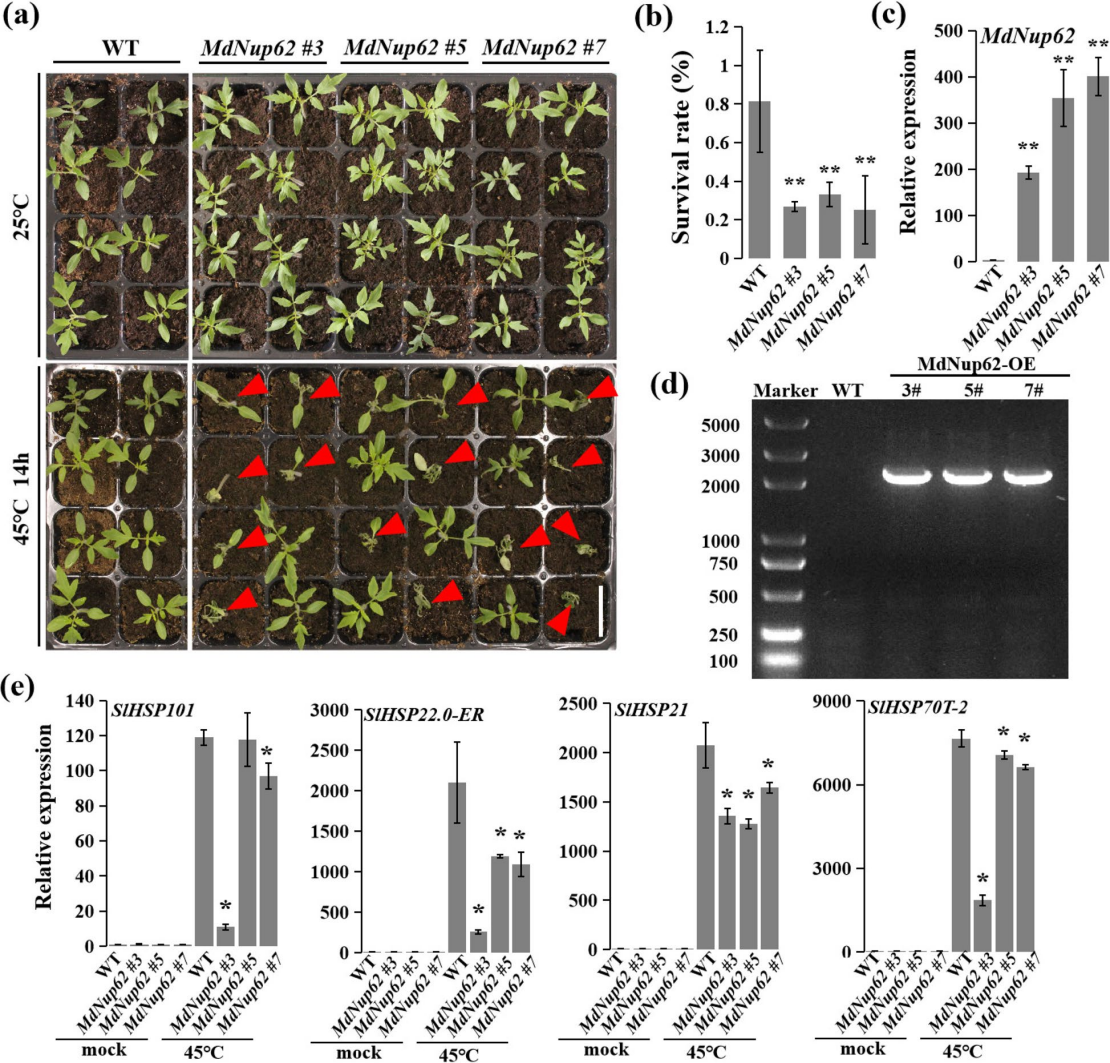
*MdNup62* reduced high-temperature resistance in tomato. **a** Phenotype of the *MdNup62*-overexpression tomato line for high-temperature resistance. Bar = 5 cm. **b** Survival rates of WT and *MdNup62*-overexpression tomato lines after the high-temperature treatment. **c** qRT-PCR analysis of *MdNup62* expression levels in tomato samples. **d** Genomic PCR analysis of *MdNup62* transgenic tomato lines. **e** Relative expression levels of high-temperature resistance-related genes (*SlHSP101*, *SlHSP22.0-ER*, *SlHSP21*, and *SlHSP70T-2*) in WT and *MdNup62*-overexpression lines at the normal temperature (22 °C) and 1 h after exposure to the high-temperature (45 °C) treatment

Because *MdNup62* was induced by high temperature, we investigated the high-temperature resistance function of *MdNup62*. OE-*MdNup62* Arabidopsis lines were subjected to a high-temperature (45 °C) treatment (Fig. 4a). Additionally, the survival rate of transgenic Arabidopsis was significantly lower than that of WT (Fig. 4b). We also performed a qRT-PCR analysis of *A. thaliana HSPs* (*AtHSP101*, *AtHSP22-ER*, *AtHSP21.0*, and *AtHSP70T-2*) (Fig. 4c). Their expression levels in transgenic Arabidopsis were reduced under high-temperature conditions. Consistently, after the heat treatment, the ROS accumulation in leaves was clear greater in OE-*MdNup62* lines compared with WT (Fig. 5a). In addition, the malondialdehyde and H_2_O_2_ levels were significantly greater than in WT (Fig. 5b, c). Moreover, the superoxide dismutase, peroxidase, and catalase activities were lower in OE-*MdNup62* lines than in WT (Fig. 5d–f). High-temperature resistance assays were carried out in transgenic tomato plants (Fig. 6a). As in transgenic *A. thaliana*, the survival rate of transgenic tomato was significantly reduced compared with WT (Fig. 6b). The presence of the transgene in OE-*MdNup62* lines was confirmed by genomic PCR, and qRT-PCR (Fig. 6c,d). The expression levels of *HSPs* (*HSP101*, *HSP22-ER*, *HSP21.0*, and *HSP70T-2*) in transgenic tomato were significantly reduced under high-temperature conditions compared with under normal growth conditions (Fig. 6e). These results indicate that *MdNup62* reduces plant high-temperature resistance.

### MdNup62-interacting protein screening

To further reveal the function of *MdNup62*, we conducted a Y2H sieve library experiment using a *MdNup62* truncated body (*MdNup62*^*508–613*^*-pGBKT7*) that is not self-activated. We identified 62 putative MdNup62-interacting proteins (Table S3). Some transcription factors were identified, such as HSFs (MdHSFA1d, MdHSFA1e, MdHSFA9, MdHSF30, MdHSF1, and MdHSF8), as well as MdMYB21, MdMYC2, MdGATA11, and MdBAK1. In addition, some enzymes and other functional genes were found. Because transcription factors that have transcriptional regulatory functions must be transported into the nucleus, and because MdNup62 has regulatory effects on the transport of the proteins, we hypothesized that MdNup62 interacts with these MdHSFs and controls their transport.

### MdNup62 interacts with MdHSFs

**Fig. 7:**
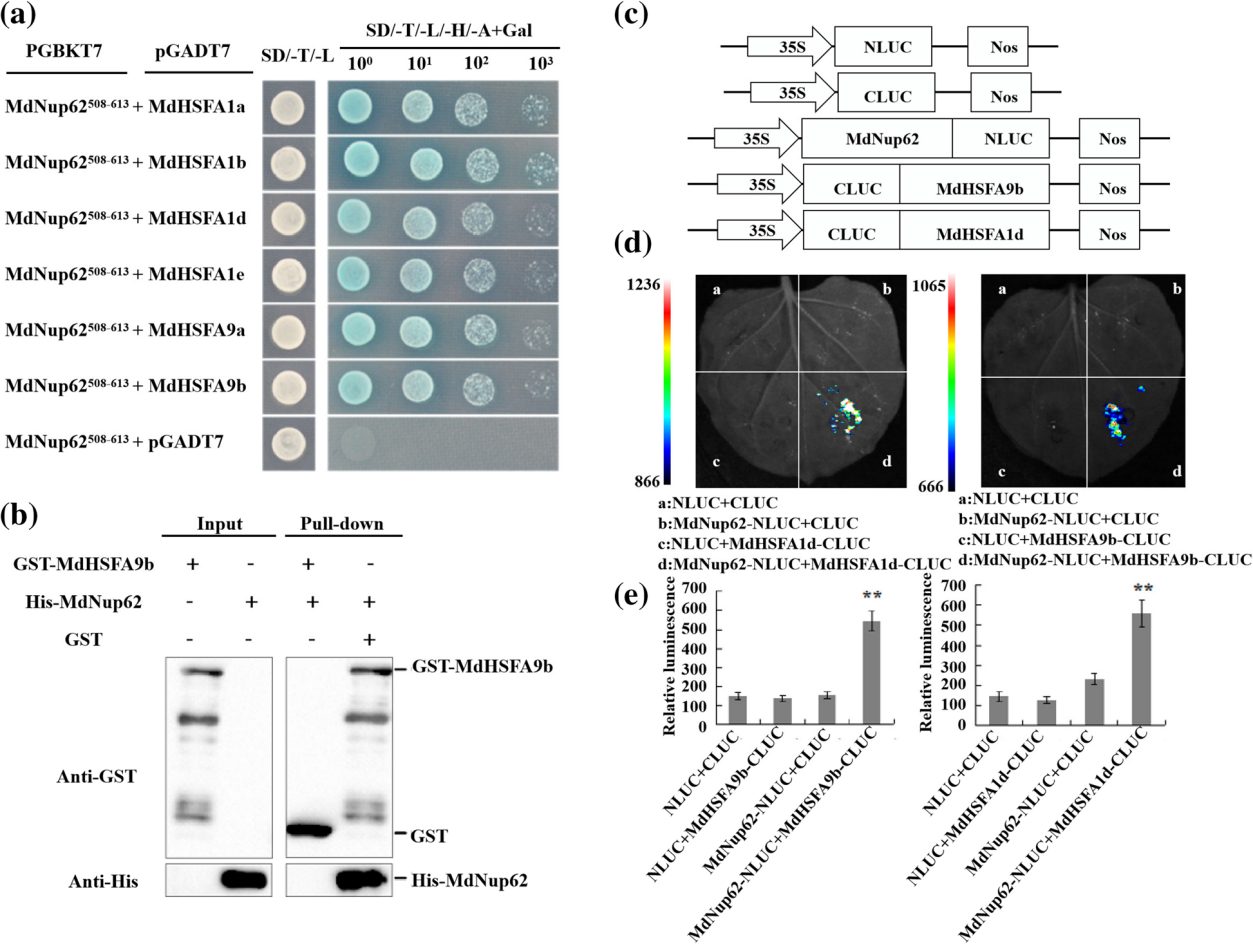
MdNup62 interacts with MdHSFs. **a** Interactions between MdNup62^508−613^ and MdHSFs (MdHSFA9a/b and MdHSFA1a/b/d/e) in Y2H assays. The *MdNup62*^*508−613*^ truncated sequence was cloned into pGBKT7, whereas *MdHSFA*s (*MdHSFA9a/b* and *MdHSFA1Da/b/d/e*) were cloned independently into the pGADT7 vector. Empty pGADT7 plus *MdNup62*.^*508−613*^-pGBKT7 was used as the control. **b** Interactions between MdNup62 and MdHSFA9b in the pull-down assay. Western blotting with a GST antibody revealed that MdNup62-HIS was pulled down by MdHSFA9b-GST. **c**–**e** Interactions between MdNup62 and both MdHSFA9b and MdHSFA1d in a luciferase (LUC) complementation experiment. Empty NLUC and empty CLUC, *MdNup62*-NLUC plus empty CLUC, empty NLUC plus *MdHSFA9b*, and *MdHSFA1d*-CLUC were used as controls

We cloned parts of the *MdHSF*s (*MdHSFA1a/b/d/e* and *MdHSFA9a/b*) independently into the pGADT7 vector and then cotransformed each with *MdNUP62*^*508–613*^*-pGBKT7*. MdNup62 interacted with these MdHSFs (Fig. 7a)*.* Additionally, we used MdHSFA9b in pull-down assays. The recombinant MdNup62-HIS fusion protein was purified with MdHSFA9b-GST, but not with GST alone (Fig. 7b). The split-LUC complementation assay revealed that the co-expression of *MdNup62-*NLUC with *MdHSFA1d*-CLUC or *MdHSFA9b*-CLUC resulted in a higher LUC activity than the other combinations (Fig. 7c–e). These results confirmed the interaction between MdNup62 and both MdHSFA1D and MdHSFA9b.

### Feature, expression, and subcellular localization analyses of *MdHSFA9b* and *MdHSFA1d*

**Fig. 8:**
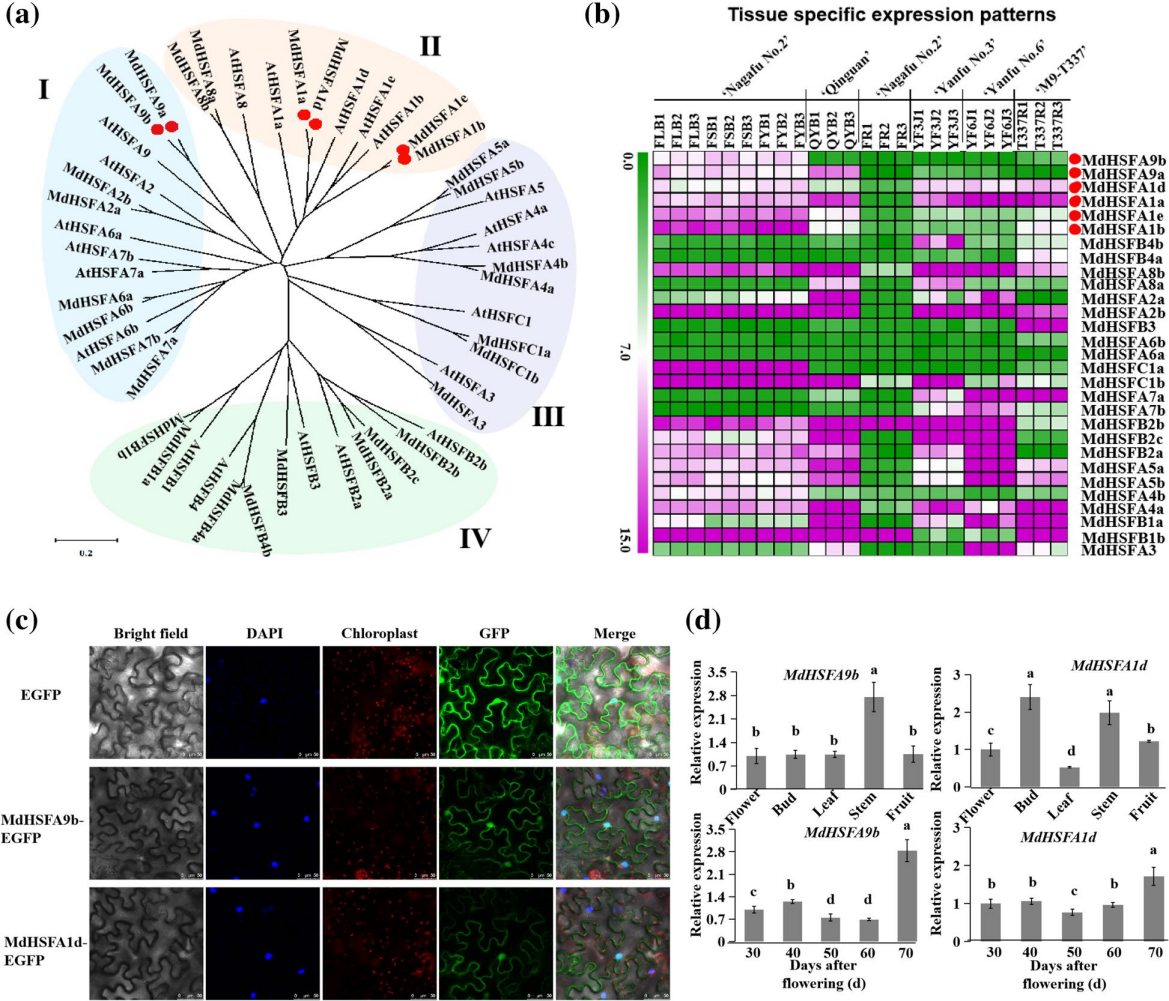
Subcellular localization and expression analyses of *MdHSFA9b* and *MdHSFA1d*. **a** Phylogenetic analysis of HSFs in Malus and Arabidopsis. **b** Tissue specific expression patterns of apple *MdHSFs* by RNA-seq. The full names of the different abbreviations are as follows, ‘Nagafu No.2’ long branches flower buds (FLB), ‘Nagafu No.2’ short branches flower buds (FSB), ‘Nagafu No.2’ axillary buds (FYB), ‘Qinguan’ axillary buds (QYB), ‘Nagafu No.2’ fruit (FR), ‘Yanfu No.3’ stem tip (YF3J), ‘Yanfu No.6’ stem tip (YF6J), and ‘M9-T337’ root (T337R). **c** Subcellular localizations of MdHSFA9b and MdHSFA1d. The upper panel shows 35S::EGFP, the middle panel shows 35S::*MdHSFA9b*-EGFP, and the lower panel shows 35S::*MdHSFA1d*-EGFP. **d** Analyses of *MdHSFA9b* and *MdHSFA1d* expression levels in diverse apple ‘Nagafu No. 2’ tissues and in different flower bud developmental stages of ‘Nagafu No. 2’

Phylogenetic tree analysis showed that Apple and Arabidopsis HSFs were divided into four groups (I, II, III, IV), with MdHSFA1a/b/d/e in groupII, and MdHSFA9a/b in groupI (Fig. 8a). We also examined the expression patterns of *MdHSFs* in different tissues of several apple varieties (Fig. 8b; Table S2). And the expression levels of *MdHSFA1a/b/d/e.*

and *MdHSFA9a/b* showed significantly higher in buds.

The subcellular localizations of MdHSFA9b and MdHSFA1d were studied by independently introducing 35S::*MdHSFA9b*-EGFP and 35S::*MdHSFA1d*-EGFP, respectively, into tobacco leaves (Fig. 8c). Tobacco leaves transformed with the empty vector 35S::EGFP served as controls. In the tobacco leaves expressing 35S::*MdHSFA9b*-EGFP and 35S::*MdHSFA1d*-EGFP, the GFP signals were observed in both the nucleus and cytoplasm, while the GFP signal was detected throughout the control tobacco leaf cells, indicating that MdHSFA9b and MdHSFA1d localized to both the nucleus and cytoplasm.

A tissue-specific expression analysis revealed that *MdHSFA1d* was expressed highest in flower buds and stems. The highest expression level of *MdHSFA9b* was in stems, but the expression levels in the other tissues were also high. Subsequently, the expression levels of *MdHSFA9b* and *MdHSFA1d* remained high during the flower bud developmental stages, while the highest was at 70 days after flowering (Fig. 8d). These results indicated that *MdHSFA9b* and *MdHSFA1d* maintained high expression levels during flower bud induction, suggesting that they may be involved in the bud differentiation of apple.

### Overexpression of *MdHSFA9b* and *MdHSFA1d* promotes flowering

**Fig. 9:**
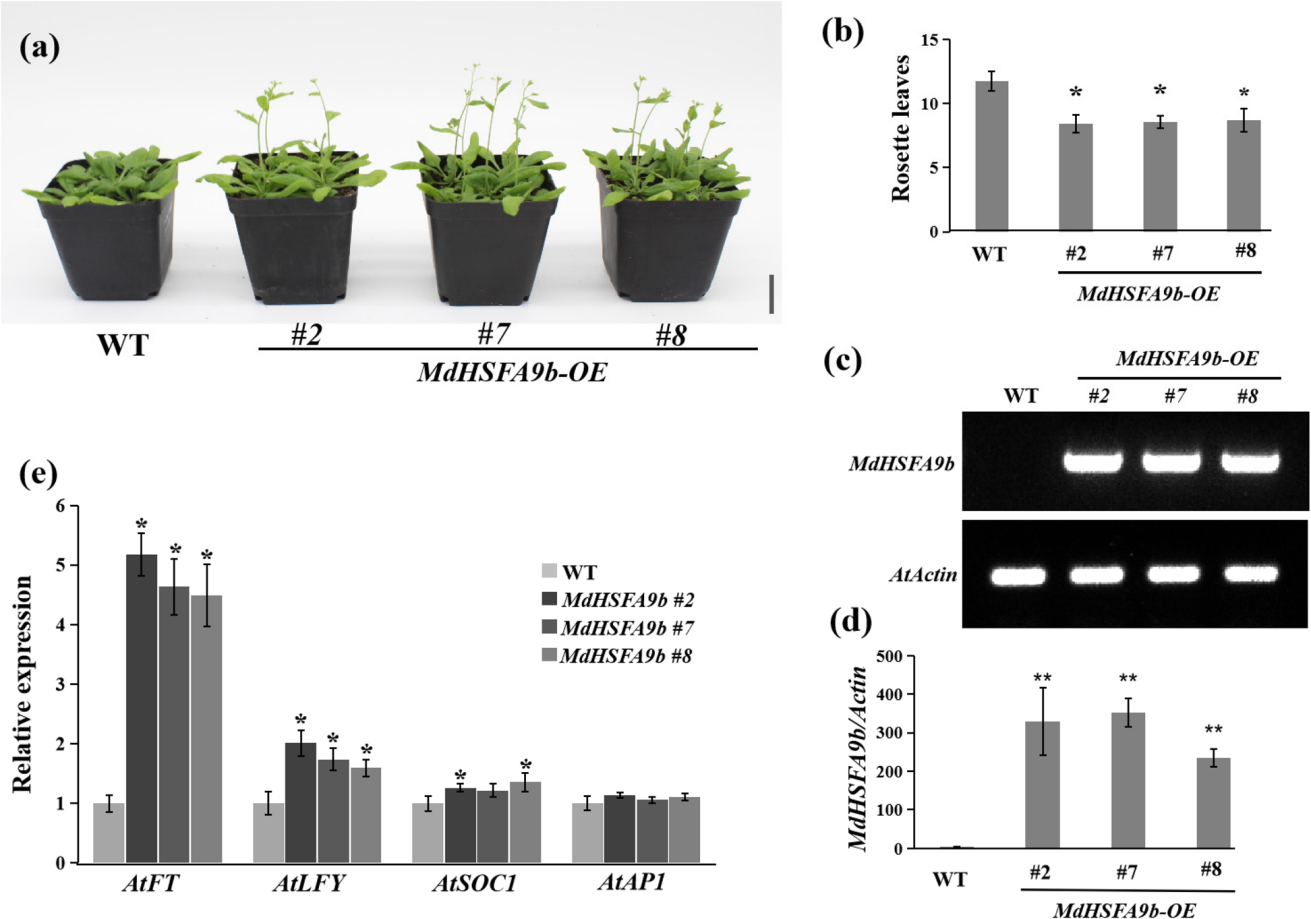
*MdHSFA9b* promotes flowering in Arabidopsis. **a** Phenotype of the *MdHSFA9b*-overexpression Arabidopsis line for flowering time. Bar = 2 cm. **b** Statistical analysis of rosette leaves of *Arabidopsis thaliana* during bolting. **c** Semi-quantitative RT-PCR analysis of *MdHSFA9b* expression in Arabidopsis samples. (d) qRT-PCR analysis of *MdHSFA9b* expression in Arabidopsis samples. **e** Relative expression levels of flowering genes (*AtFT*, *AtLFY*, *AtSOC1*, and *AtAP1*) in WT and *MdHSFA9b*-overexpression lines

To verify the flowering phenotype of HSFs, we performed Agrobacterium-mediated genetic transformations of *MdHSFA9b* and *MdHSFA1d* into *A. thaliana*. Like OE-*MdNup62*, OE-*MdHSFA9b* and OE-*MdHSFA1d* lines flowered significantly earlier than WT (Figs. 9a and S3a). Additionally, they also had significantly fewer rosette leaves than WT during bolting (Figs. 9b and S3b). We also performed genomic PCR (Figure S2b, c), semi-quantitative RT-PCR (Figs. 9c and S3c), and qRT-PCR (Figs. 9d and S3d) to confirm the presence of the transgene in the OE-*MdHSFA9b* and OE-*MdHSFA1d* lines. The transcript levels of *AtFT*, *AtLFY*, and *AtSOC1* were significantly increased in OE-*MdHSFA9b* and OE-*MdHSFA1d* lines compared with WT (Figs. 9e and S3e).

### Overexpression of *MdHSFA9b* and *MdHSFA1d* enhances high-temperature resistance

**Fig. 10:**
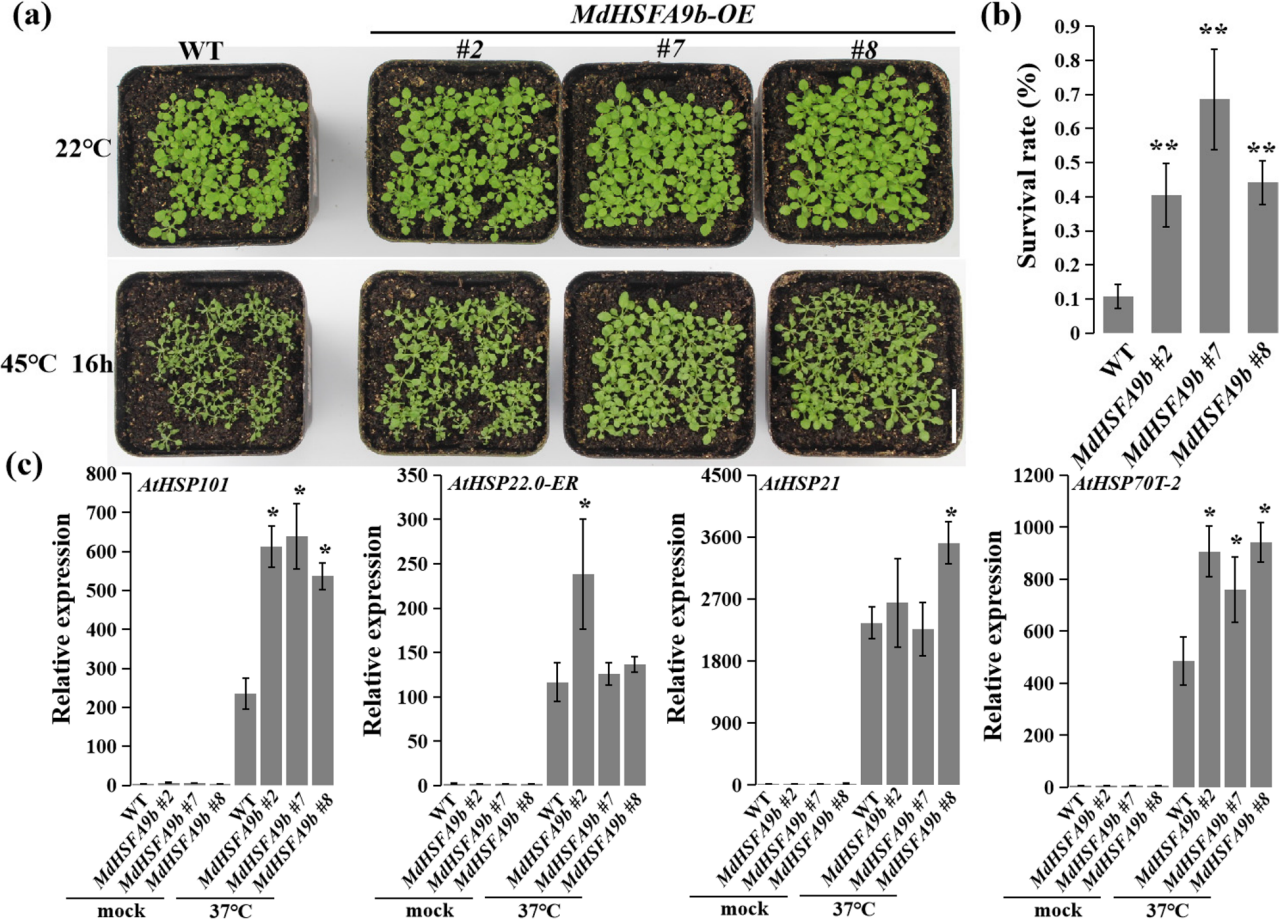
*MdHSFA9b* enhanced high-temperature resistance in Arabidopsis. **a** Phenotype of the *MdHSFA9b*-overexpression Arabidopsis line for high-temperature resistance. Bar = 2 cm. **b** Survival rates of WT and *MdHSFA9b*-overexpression Arabidopsis lines after the high-temperature treatment. **c** Relative expression levels of high-temperature resistance-related genes (*AtHSP101*, *AtHSP22.0-ER*, *AtHSP21*, and *AtHSP70T-2*) in WT and *MdHSFA9b*-overexpression lines at the normal temperature (22 °C) and 1 h after exposure to the high-temperature (37 °C) treatment

To study the high-temperature resistance phenotypes of *MdHSFA9b* and *MdHSFA1d*, we also exposed OE-*MdHSFA9b* and OE-*MdHSFA1d* transgenic plants, respectively, to high-temperature (45 °C) conditions (Figs. 10a and S4a). The survival rates of OE-*MdHSFA9b* and OE-*MdHSFA1d* lines were significantly greater than that of WT (Figs. 10b and S4b). Consistently, the ROS accumulation in leaves decreased in transgenic plants after the high-temperature treatment (Figure S5a, b). We also performed a qRT-PCR analysis of *A. thaliana HSPs* (*AtHSP101*, *AtHSP22-ER*, *AtHSP21.0*, and *AtHSP70T-2*) (Figs. 10c and S4c), and their expression levels in transgenic *A. thaliana* increased under high-temperature conditions compared with under normal growth conditions. These results indicated that *MdHSFA9b* and *MdHSFA1d* enhance plant high-temperature resistance.

## Discussion

Plant flowering has always been an important topic in crop and horticultural sciences, and issues with apple flowering have long hindered the development of the apple industry in China [1, 2]. The Nups control protein transport between the nucleus and cytoplasm, and they participate in a variety of biological processes, including flowering [19, 20]. In *A. thaliana*, Nup96 promotes the stability of HOS1, and HOS1 conjugates and degrades CO, then promotes *FLC* expression, leading to delayed flowering. In addition, HOS1 increases the stability of Nup96 and thus maintains this regulatory pathway to control the flowering time [23, 26]. Mutations in *Nup54*, *Nup58*, *Nup62*, *Nup136*, and *Nup160* have resulted in a prominent earlier flowering phenotype compared with WT [16, 25]. In the present study, *MdNup62* maintained a high expression level during flower development. To verify the flowering function of *MdNup62*, we determined the flowering phenotypes of OE-*MdNup62 A. thaliana* lines. Interestingly, the phenotypes of the overexpression lines were consistent with Arabidopsis deletion mutants and showed obvious early flowering. Previous studies found that both *Nup62* deletion mutants and overexpression strains of Arabidopsis have increased the sensitivities to auxin, indicating that the overexpression does not result in a functional gain, but rather a functional loss, like the mutant [51]. Therefore, the overexpression of *MdNup62* in this study may also result in a functional loss. However, *MdNup62* is involved in the flowering pathway.

With global warming, extreme high-temperatures will occur more frequently, which will seriously affect the normal growth and development of plants [5, 6]. And *Nups* are involved in temperature-stress responses. *HOS1* and *Nup160* were reported to be involved in cold resistance [27, 28]. Nup85 and Nup133 control mRNA output only under warm conditions and are more sensitive to transcription factor localization at warm temperatures [20]. In this study, *MdNup62* responded to high-temperature stress in apple. However, OE-*MdNup62* lines had reduced high-temperature resistance in both Arabidopsis and tomato. By analysing the relative expression levels of *HSPs* (*HSP101*, *HSP22-ER*, *HSP21.0*, and *HSP70T-2*) in transgenic plants, we found no obvious correlations between OE-*MdNup62* lines and WT at a normal growth temperature, but OE-*MdNup62* lines had significantly lower *HSP* expression levels than WT under high-temperature conditions.

In plants, Nup-interacting proteins have been studied [17, 18, 26], and some potential Nup85-interacting proteins have been identified by immunoprecipitation and subsequent mass spectrometry in Arabidopsis, such as the Nup107–160 subcomplex (Nup160, Nup133, Nup43, Nup96, Nup107, Seh1, and Sec13), several mediator subunits (MED16, MED14, and MED18), HOS1, and Sec13A. The interactions between Nup85 and three proteins, HOS1, Sec13A, and MED18, have been confirmed. Additionally, a direct interaction between Nup96 and HOS1 in Arabidopsis has also been reported [26]. In our previous study, the interaction between MdNup54 and MdNup62 was confirmed in apple [17]. However, there are no reports of direct interactions between transcription factors and Nups in plants. We previously identified an interaction between apple MdNup54 and MdKNAT4/6 using a yeast double-hybridization test, but further verification is needed [17]. In this study, we verified direct interactions between MdNup62 and MdHSFs, indicating that the Nups may directly recognize related transcription factors and thus regulate their transport. This provides a new direction of study for Nups.

Because of the early flowering of OE-*MdNup62* Arabidopsis lines, MdHSFs that interact with MdNup62 may be also involved in the flowering pathway. Consistent with this conjecture, some HSFs are associated with flowering [49, 50]. HSFA1E and HSFA4C directly target and positively regulate the flowering gene *SOC1* in lettuce [49]. Arabidopsis HSFA2 directly targets and promotes the expression of *REF6*, and the *REF6–HSFA2* loop directly targets and activates *HTT5*, which coordinates early flowering [50]. In this study, we found that *MdHSFA9b* and *MdHSFA1d* maintained high expression levels during flower bud induction. Additionally, OE-*MdHSFA9b* and OE*-MdHSFA1d* Arabidopsis lines flower significantly earlier than WT. This suggests that *MdHSFA9b* and *MdHSFA1d* promote plant flowering. *MdNup62*, *MdHSFA9b*, and *MdHSFA1d* share the same flowering phenotype, possibly because the overexpression of *MdNup62* fosters HSF accumulation in the nucleus, promoting the expression of downstream flowering-related genes and advancing flowering.

HSFs play important roles in regulating plant resistance to high temperatures. *HSFA1* positively regulates the heat tolerance of tomato, the expression of *HSFA2* is dependent on HsfA1, and the thermotolerance of the posttranscriptional silencing of the *HsfA1* gene in protoplasts can be restored by plasmid-borne *HsfA2* [52]. HSFA1d and HSFA1e activate *HsfA2* transcription, and a double knockout of *HSFA1d* and *HSFA1e* impairs tolerance to heat-shock stress [43]. In *Medicago truncatula*, *HSFA9* plays important roles in thermotolerance [53]. In the current study, we obtained similar results for *MdHSFA9b* and *MdHSFA1d*. The expression levels of *HSPs* in the two overexpression Arabidopsis lines were significantly greater than in WT, and both lines had enhanced high-temperature resistance levels. Like the flowering and auxin phenotypes [51], the opposite phenotypes between OE-*MdNup62* and OE-*MdHSFA9b,* OE*-MdHSFA1d* indicates that the overexpression of *MdNup62* may also result in a lack of function under heat-stress conditions. Similar to the results of this study, Zhang et al. (2020) found that *nup85* and *nup133* increase the ubiquitous protoplast (nucleus and cytosol) signals of IAA17 and PIF4 at 28 °C compared with at 22 °C. Furthermore, the *nup96* and *hos1* mutants show significant increases in the ubiquitous localizations of IAA17 and PIF4 signals at 28 °C (72% and 66%, respectively) compared with 22 °C (40% and 49%, respectively)[20]. Thus, the nuclear accumulations of the IAA17and PIF4 proteins in *nup85*, *nup96*, *nup133*, and *hos1* are reduced compared with WT, and the defects are more severe at 28 °C. Therefore, we hypothesized that the transport of MdHSFA9b, MdHSFA1d, and other MdHSFs is inhibited in OE-*MdNup62* lines at high temperatures, resulting in the inhibition of the transcription of downstream *HSP*s, which further reduces high-temperature resistance.

**Fig. 11:**
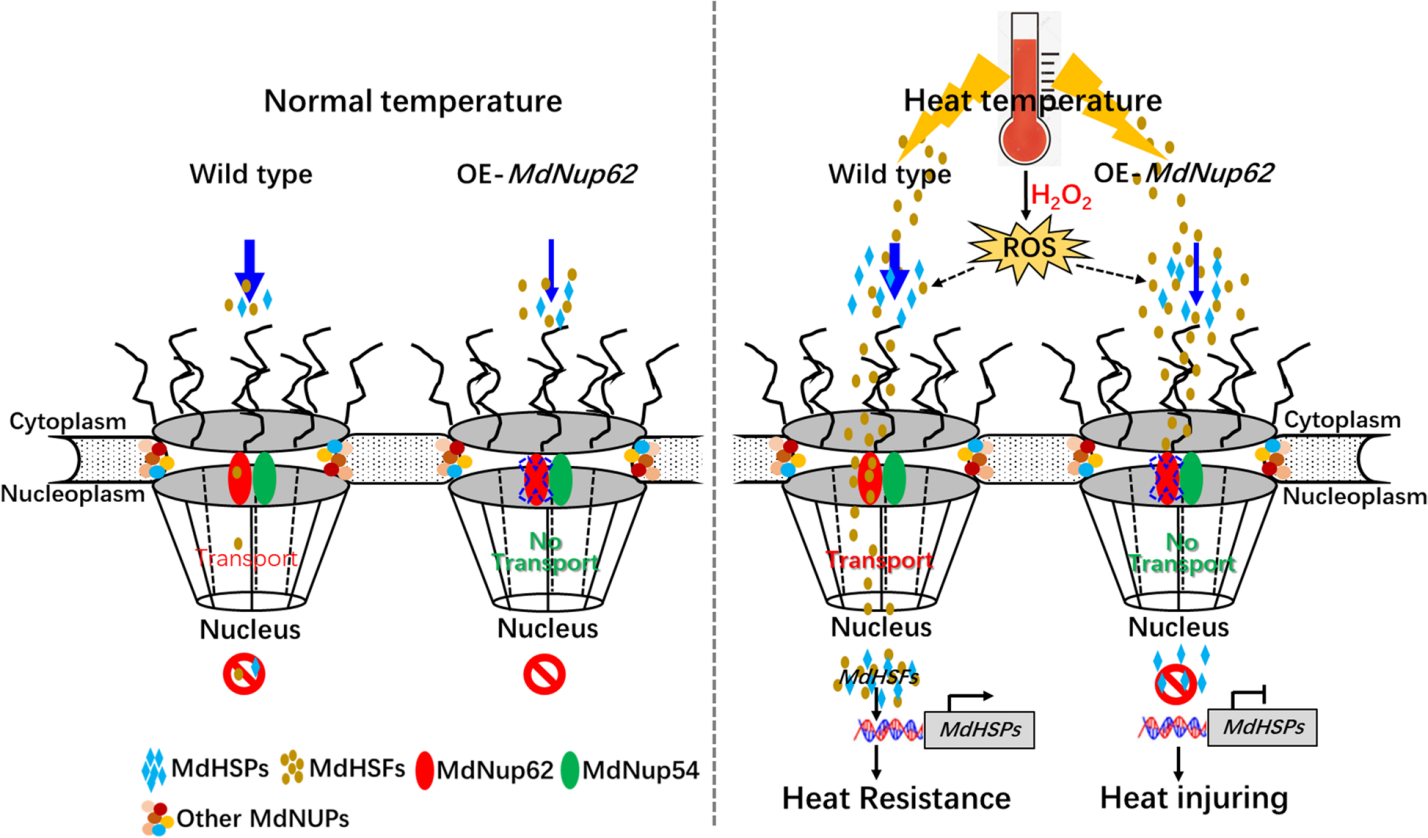
Model of MdNup62 interactions with MdHSFs involved in heat-stress tolerance in apple

On the basis of these findings, we constructed a hypothetical model of *MdNup62-*related pathways involved in high-temperature resistance (Fig. 11). At normal temperature, apple *MdHSFs* were not induced, and not much transported into nucleus that cannot lead to up-regulate expression of *MdHSPs* in WT and OE-*MdNup62*. However, at high temperature, apple *MdHSFs* were significantly induced, and then transported into the nucleus through NPC channels to promote the expression of *MdHSPs* in WT, in which enhanced high-temperature resistance. But for OE-*MdNup62* lines, the structure of the apple NPC changed, and blocked the transport of high temperature induced MdHSFs into the nucleus that cannot induce much *MdHSPs* expression causing heat injuring (Fig. 11). Additionally, OE-*MdNup62*, OE-*MdHSFA9b* and OE-*MdHSFA1d* lines showed significant early flowering phenotype compared with WT (Fig. 3, 9; Figure S3).

In conclusion, temperature is an important factor affecting flowering. With global warming, apple flowering will occur earlier, increasing the risk of chilling-related injury. Moreover, extreme hot weather is also occurring frequently. Both climatic conditions seriously affect the development of the apple industry. MdNup62 interacts with MdHSFs to regulate flowering and heat-resistance pathways in plants. Thus, both *MdNup62* and the *MdHSFs* regulate flowering and respond to temperature changes. This research provides a theoretical reference for managing the impact of global warming on the apple industry.

## Materials and methods

### Plant materials and growth conditions

The plant materials were 6-year-old apple trees (‘Fuji’ /T337/*Malus robusta* Rehd.) growing in the experimental orchard of the Horticulture College of Northwest A & F University (108°04′ E, 34°16′ N). We collected new shoots (2–3 mm in diameter) near the tips, fully expanded leaves near buds, flower buds, blooming flowers, and young fruit, which were immediately frozen in liquid nitrogen and stored at − 80 °C for later use.

The ‘Fuji’ plants were grown on MS medium containing 0.1 mg·L^−1^ indolebutyric acid and 0.6 mg·L^−1^ 6-benzylaminopurine under long-day conditions (16 h-light/8 h-dark) at 24 °C and were subcultured every 45 days. Arabidopsis plants(‘Columbia’) were grown under long-day conditions (16 h-light/8 h-dark) at 22 °C. Tomato plants (‘Ailsa Craig’) were grown under long-day conditions (16 h-light/8 h-dark) at 25 °C. And the arabidopsis and tomato seeds were previously preserved in our laboratory.

### Heat map, protein alignment, and phylogenetic analysis

Based on RNA-seq data of our laboratory, the heat map of apple different tissues was constructed using MeV (Multiple Experiment Viewer) software. A protein sequence alignment of Nup62 from six Rosaceae plants was performed using DNAMAN software. The Nup62 protein sequences were obtained from the GDR database (https://www.rosaceae.org/). The phylogenetic tree was constructed using MEGA-X software.

### RNA extraction and qRT-PCR analysis

Total RNA was extracted from apple trees, Arabidopsis seedlings, tomato seedlings, and apple seedlings using an RNA Plant Plus Reagent Kit (TIANGEN, Beijing, China). The RNA was used as the template to synthesize cDNA with a PrimeScript RT Reagent Kit (Takara, Shiga, Japan). The qRT-PCR analysis was conducted on a StepOnePlus Real-Time PCR System (Thermo Fisher Scientific, USA). The reaction solution contained 10 μL SYBR Green I Master Mix (CWBIO, Beijing, China), 0.5 μmol·L^−1^ primers (SANGON BIOTECH, Shanghai, China), and 1 μL each template in a total volume of 20 μL. The PCR program was as follows: 95 °C for 3 min; 40 cycles of 94 °C for 15 s, 60 °C for 20 s, and 72 °C for 15 s. All the samples were analysed with three biological replicates, each comprising three technical replicates. Relative gene expression levels were calculated in accordance with the 2^−ΔΔCt^ method [54]. The primers used for qRT‐PCR (Table S4) were synthesized by the Sangon Biotechnology Co. Ltd. (Shanghai, China).

### Subcellular localization

The open reading frames (ORFs) of the *MdNup62*, *MdHSFA1d*, and *MdHSFA9b* genes were inserted independently into the pCAMBIA2300-EGFP vector to generate the 35S::*MdNup62*-EGFP, 35S::*MdHSFA1d*-EGFP, and 35S::*MdHSFA9b*-EGFP recombinant plasmids, respectively. These recombinant plasmids were inserted independently into *Agrobacterium tumefaciens* strain GV3101 cells. The GV3101 cells containing these recombinant plasmids were then infiltrated into tobacco leaves. GV3101 cells containing the pCAMBIA2300-EGFP vector (35S::EGFP) served as the control. After an additional 3 days of growth in the dark, green fluorescent protein (GFP) signals in transformed tobacco leaves were detected using a Leica TCS SP8 SR Laser Scanning Confocal Microscope (Leica, Germany).The primers used are listed in Table S5.

### Genetic transformation

The genetic transformations were performed in accordance with published methods for Arabidopsis [55] and tomato (‘Ailsa Craig’) [56] plants. The transgenic Arabidopsis and tomato lines were selected on MS plates supplemented with 50 mg·L^−1^ and 100 mg·L^−1^ kanamycin, respectively.

### Yeast two-hybrid (Y2H) assay

The *MdNup62*^508–613^ truncated sequence was cloned into the pGBKT7 vector to generate the *MdNup62*^508–613^-pGBKT7 recombinant plasmid. The *MdHSFAs*’ ORFs were inserted individually into the pGADT7 vector to generate the *MdHSFAs*-pGADT7 recombinant plasmids. The recombinant plasmids were inserted into Gold Yeast Two-Hybrid cells, which were then grown on a selective medium. The primers used are listed in Table S5.

### Split luciferase (LUC) complementation

The full-length *MdHSFA1d* and *MdHSFA9b* coding sequences were cloned independently into the CLUC vector, while *MdNup62* was cloned into the NLUC vector. The split-LUC complementation assay was performed with tobacco leaves. The reconstituted LUC activity was detected in the dark using a Princeton Lumazone Pylon 2048B cooling camera (Princeton, USA). The LUC activity was quantified using the Dual-Luciferase Reporter Assay System (Promega, USA). The primers used are listed in Table S5.

### Pull-down assays

The ORFs of *MdNup62* and *MdHSFA9b* were cloned into the pET-28a and pGEX-6p-1 vectors, respectively, and subsequently overexpressed independently in *Escherichia coli* BL21(DE3) (Transgene). The pull-down assays were conducted using the His-Tagged Protein Purification Kit (Clontech) in accordance with the manufacturer’s instructions. The primers used are listed in Table S5.

### Heat-tolerance assays

The ‘Fuji’ plants at 30 days after propagation were used for the 45 °C heat treatment. We collected leaf samples before and at 1, 3, and 6 h after the treatment. The samples were immediately frozen in liquid nitrogen and stored at − 80 °C for later use.

Two-week-old transgenic Arabidopsis and 3-week-old transgenic tomato were used for the heat treatment in an artificial climate chamber. OE-*MdNup62 A. thaliana* lines were subjected to 45 °C for 12 h, and OE-*MdHSFA9b* and OE-*MdHSFA1d A. thaliana* lines were subjected to 45 °C for 16 h. OE-*MdNup62* tomato lines were subjected to 45 °C for 14 h.

### Evaluation of stress tolerance

The superoxide dismutase, peroxidase, and catalase activities and the malondialdehyde and H_2_O_2_ levels were detected using the corresponding Suzhou Comin Biotechnology test kits (Suzhou Comin Biotechnology Co., Ltd, Suzhou, China). The presence of O^2−^ in leaf samples was determined by staining with nitro blue tetrazolium.

### Statistical analyses

Statistical analyses were performed using SPSS software. Data are reported as means ± SDs. Asterisks (*) indicate significant differences between treatments as assessed by Student’s t-test at *P* < 0.05 (*) and *P* < 0.01 (**). Different lowercase letters above the bars indicate significant differences (*P* < 0.05, Tukey’s test).

## Supplementary Information

**Additional file 1: Table S1.** Expression of NPC components in different tissues ofseveral apple varieties. **Table S2.** Expression of *MdHSFs* in different tissues of several apple varieties. **Table S3.** MdNup62 yeast double-hybridization screening results. **TableS4.** Primersused for qRT-PCR. **TableS5.** Primers used for plasmid construction. **Figure S1.** Interactions between MdNup62 and MdNup54 in aluciferase (LUC) complementation experiment. **Figure S2.**GenomicPCRanalyses of *MdNup62 *(a), *MdHSFA9b *(b), and* MdHSFA1d *(c) in transgenic Arabidopsis lines. **Figure S3.** *MdHSFA1d* promotes flowering inArabidopsis. **FigureS4.** *MdHSFA1d* enhanced high-temperatureresistance in Arabidopsis. **Figure S5.** Changes inthe levels of accumulated ROS in Arabidopsis leaves under heat-stressconditions. **Figure S6.** Schematic diagram of vector. **FigureS7.** Original image ofnucleic acid electrophoresis. **Figure S8.** Original image of Figure 7b.

## Data Availability

All data generated or analysed during the current study are available in this article and its supplementary information files. Gene sequences can be downloaded at NCBI database (https://www.ncbi.nlm.nih.gov/). And the GenBank accession number of MdNup62 is MT102240, MdHSFA9a is ON364334, MdHSFA9b is ON364335, MdHSFA1a is ON364336, MdHSFA1b is ON364337, MdHSFA1d is ON364338, MdHSFA1e is ON364339, and MdNup54 is MT102239.

## Abbreviations

HSF: Heat shock factor
MS: Murashige and skoog
NPC: Nuclear pore complex
OE: Overexpression
ORF: Open reading frame
PCR: Polymerase chain reaction
qRT-PCR: Quantitative real-time PCR
ROS: Reactive oxygen species
WT: Wild type
