# Supplementary material for: MdNup62 interactions with MdHSFs involved in flowering and heat-stress tolerance in apple
Source: BMC Plant Biol. 2022 Jul 4;22:317. doi: 10.1186/s12870-022-03698-3 (PMC9251929; doi:10.1186/s12870-022-03698-3)
Supplement: Supplementary file 1 — Additional file 1: Table S1. Expression of NPC components in different tissues ofseveral apple varieties. Table S2. Expression of MdHSFs in different tissues of several apple varieties. Table S3. MdNup62 yeast double-hybridization screening results. TableS4. Primersused for qRT-PCR. TableS5. Primers used for plasmid construction. Figure S1. Interactions between MdNup62 and MdNup54 in aluciferase (LUC) complementation experiment. Figure S2.GenomicPCRanalyses of MdNup62 (a), MdHSFA9b (b), and MdHSFA1d (c) in transgenic Arabidopsis lines. Figure S3. MdHSFA1d promotes flowering inArabidopsis. FigureS4. MdHSFA1d enhanced high-temperatureresistance in Arabidopsis. Figure S5. Changes inthe levels of accumulated ROS in Arabidopsis leaves under heat-stressconditions. Figure S6. Schematic diagram of vector. FigureS7. Original image ofnucleic acid electrophoresis. Figure S8. Original image of Figure 7b. [file 12870_2022_3698_MOESM1_ESM.docx]

**MdNup62 interactions with MdHSFs involved in flowering and heat-stress tolerance in apple**

Chenguang Zhang†, Na An†, Peng Jia†, Wei Zhang, Jiayan Liang, Hua Zhou, Dong Zhang, Juanjuan Ma, Caiping Zhao, Mingyu Han, Xiaolin Ren, Libo Xing*

^1^College of Horticulture, Northwest A&F University, 712100 Yangling, Shaanxi, P. R. China

**^†^Equal contributors**

***Corresponding author:**

Libo Xing

E-mail: [libo_xing@nwsuaf.edu.cn](mailto:libo_xing@nwsuaf.edu.cn) ;

Tel.: +8615129227289;

ORCID: https://orcid.org/0000-0002-8918-7128;

Address: 3 Taicheng Road, Yangling 712100, Shaanxi, P. R. Chin

Run title: MdNup62 interactions with MdHSFs regulate flowering and heat-stress tolerance

**Table S1. Expression of NPC components in different tissues of several apple varieties**

|  |  |  | **‘Nagafu No.2’** | | | | | | | | |  | **‘Qinguan’** | | |  | **‘Nagafu No.2’** | | |  | **‘Yanfu No.3’** | | |  | **‘Yanfu No.6’** | | |  | **‘M9-T337’** | | |
| --- | --- | --- | --- | --- | --- | --- | --- | --- | --- | --- | --- | --- | --- | --- | --- | --- | --- | --- | --- | --- | --- | --- | --- | --- | --- | --- | --- | --- | --- | --- | --- |
| **Subcomplex** | **Gene** | **Gene ID** | **FLB1** | **FLB2** | **FLB3** | **FSB1** | **FSB2** | **FSB3** | **FYB1** | **FYB2** | **FYB3** |  | **QYB1** | **QYB2** | **QYB3** |  | **FR1** | **FR2** | **FR3** |  | **YF3J1** | **YF3J2** | **YF3J3** |  | **YF6J1** | **YF6J2** | **YF6J3** |  | **T337R1** | **T337R2** | **T337R3** |
| **Nup62 subcomplex** | **MdNup54** | MD16G1117500 | 4.18 | 4.80 | 4.54 | 4.54 | 5.31 | 4.74 | 4.63 | 4.77 | 4.26 |  | 3.43 | 3.84 | 3.13 |  | 0.47 | 0.24 | 0.66 |  | 8.81 | 8.81 | 7.61 |  | 8.86 | 8.61 | 8.20 |  | 5.69 | 5.88 | 5.13 |
|  | **MdNup62** | MD07G1110700 | 15.93 | 16.76 | 15.71 | 17.82 | 16.65 | 18.51 | 17.45 | 18.36 | 16.89 |  | 16.28 | 17.09 | 17.55 |  | 9.84 | 9.53 | 9.60 |  | 18.68 | 18.08 | 18.42 |  | 22.01 | 20.31 | 20.92 |  | 16.77 | 17.75 | 18.30 |
| **Nup93 subcomplex** | **MdNup35a** | MD09G1205800 | 14.05 | 13.68 | 12.14 | 12.80 | 14.05 | 13.94 | 14.93 | 15.74 | 15.08 |  | 5.66 | 6.48 | 6.57 |  | 0.00 | 0.00 | 0.00 |  | 11.28 | 10.57 | 11.24 |  | 9.27 | 9.40 | 8.90 |  | 10.52 | 11.28 | 9.18 |
|  | **MdNup35b** | MD17G1186700 | 13.50 | 10.44 | 13.21 | 14.70 | 13.55 | 15.30 | 15.90 | 13.03 | 15.33 |  | 8.93 | 8.43 | 10.16 |  | 2.88 | 5.71 | 3.94 |  | 15.21 | 16.83 | 17.78 |  | 14.89 | 16.81 | 13.33 |  | 9.73 | 8.95 | 10.32 |
|  | **MdNup93a** | MD12G1080600 | 9.07 | 9.34 | 8.83 | 9.41 | 8.64 | 9.56 | 10.04 | 8.44 | 9.10 |  | 18.71 | 20.51 | 18.86 |  | 9.07 | 8.39 | 8.89 |  | 17.51 | 14.67 | 15.65 |  | 13.87 | 13.60 | 12.94 |  | 13.40 | 13.92 | 14.00 |
|  | **MdNup93b** | MD14G1076700 | 8.15 | 7.79 | 8.34 | 7.94 | 8.18 | 8.90 | 8.90 | 8.99 | 9.17 |  | 6.59 | 6.54 | 6.79 |  | 1.37 | 1.17 | 1.24 |  | 10.81 | 10.21 | 9.88 |  | 8.96 | 7.16 | 7.11 |  | 3.46 | 4.32 | 4.07 |
|  | **MdNup155** | MD13G1020400 | 10.35 | 10.07 | 9.20 | 10.35 | 10.43 | 9.86 | 11.67 | 11.89 | 11.14 |  | 5.63 | 5.62 | 6.48 |  | 0.00 | 0.00 | 0.00 |  | 8.26 | 8.33 | 7.47 |  | 6.17 | 5.85 | 5.15 |  | 7.02 | 7.04 | 6.35 |
|  | **MdNup205** | MD02G1032900 | 9.22 | 11.82 | 8.98 | 10.62 | 9.20 | 10.79 | 12.95 | 12.97 | 10.34 |  | 6.22 | 6.41 | 6.61 |  | 3.23 | 3.22 | 3.04 |  | 10.57 | 10.30 | 9.22 |  | 10.54 | 9.02 | 8.08 |  | 8.03 | 7.60 | 7.59 |
| **Nup107 subcomplex** | **MdNup43** | MD10G1281400 | 10.70 | 12.02 | 10.48 | 13.63 | 14.40 | 11.12 | 12.27 | 11.25 | 12.08 |  | 10.25 | 9.45 | 9.80 |  | 11.86 | 10.51 | 10.98 |  | 22.53 | 20.56 | 21.52 |  | 20.15 | 21.88 | 17.41 |  | 16.60 | 16.31 | 17.28 |
|  | **MdNup85** | MD15G1093200 | 15.82 | 16.05 | 15.61 | 16.45 | 17.55 | 17.21 | 17.28 | 17.94 | 17.33 |  | 17.86 | 18.49 | 18.96 |  | 8.14 | 8.92 | 8.94 |  | 17.18 | 17.32 | 16.51 |  | 15.73 | 13.97 | 13.19 |  | 15.95 | 15.66 | 16.16 |
|  | **MdNup96a** | MD08G1215300 | 9.00 | 9.12 | 8.94 | 8.76 | 8.87 | 9.75 | 10.24 | 10.16 | 10.44 |  | 5.66 | 5.80 | 5.82 |  | 6.81 | 6.96 | 6.58 |  | 6.81 | 6.61 | 5.96 |  | 3.31 | 3.22 | 2.78 |  | 6.78 | 5.54 | 5.31 |
|  | **MdNup96b** | MD15G1399200 | 5.05 | 5.68 | 5.08 | 4.29 | 5.11 | 5.50 | 5.60 | 6.07 | 6.45 |  | 2.02 | 2.10 | 2.13 |  | 2.79 | 0.60 | 2.33 |  | 5.91 | 7.09 | 5.42 |  | 3.47 | 3.10 | 2.92 |  | 7.94 | 7.29 | 7.57 |
|  | **MdNup107a** | MD09G1178100 | 5.76 | 5.81 | 5.58 | 6.64 | 6.53 | 7.55 | 6.42 | 6.47 | 7.55 |  | 2.49 | 2.64 | 2.28 |  | 0.00 | 0.00 | 0.00 |  | 8.17 | 8.17 | 7.82 |  | 8.63 | 7.52 | 7.16 |  | 4.54 | 4.05 | 4.18 |
|  | **MdNup107b** | MD17G1148600 | 9.33 | 9.76 | 8.28 | 9.65 | 9.14 | 9.51 | 10.42 | 10.95 | 10.37 |  | 7.30 | 7.38 | 7.35 |  | 0.00 | 0.00 | 0.00 |  | 10.28 | 10.58 | 9.70 |  | 7.75 | 7.07 | 6.51 |  | 11.17 | 11.05 | 9.96 |
|  | **MdNup133** | MD17G1113600 | 12.02 | 11.78 | 11.46 | 10.78 | 11.06 | 12.30 | 12.90 | 13.71 | 13.72 |  | 3.98 | 3.81 | 3.98 |  | 3.49 | 3.41 | 3.60 |  | 6.44 | 6.79 | 6.00 |  | 1.90 | 1.85 | 1.96 |  | 4.81 | 4.77 | 4.04 |
|  | **MdNup160** | MD10G1009800 | 11.64 | 12.29 | 10.20 | 12.90 | 11.35 | 12.79 | 14.63 | 13.17 | 12.25 |  | 13.46 | 13.71 | 14.17 |  | 3.99 | 3.71 | 3.96 |  | 10.24 | 10.03 | 10.00 |  | 8.37 | 7.23 | 7.21 |  | 11.69 | 11.23 | 10.23 |
|  | **MdSec13a** | MD09G1041000 | 92.20 | 82.31 | 92.56 | 98.27 | 109.51 | 105.45 | 98.23 | 96.62 | 105.96 |  | 42.44 | 39.68 | 42.56 |  | 46.21 | 44.03 | 45.67 |  | 62.61 | 63.32 | 60.10 |  | 59.89 | 57.42 | 48.87 |  | 45.40 | 43.26 | 40.43 |
|  | **MdSec13b** | MD17G1042300 | 33.69 | 30.55 | 32.98 | 30.72 | 31.64 | 28.96 | 33.71 | 32.49 | 30.66 |  | 20.17 | 20.86 | 19.41 |  | 0.02 | 2.56 | 2.97 |  | 25.72 | 27.20 | 25.91 |  | 27.03 | 23.48 | 29.86 |  | 21.04 | 23.06 | 21.46 |
|  | **MdSeh1a** | MD06G1103700 | 13.39 | 14.90 | 13.27 | 14.55 | 12.41 | 13.05 | 16.42 | 14.52 | 15.63 |  | 7.43 | 9.84 | 10.22 |  | 7.80 | 9.43 | 9.78 |  | 16.73 | 18.03 | 13.27 |  | 12.62 | 11.58 | 12.26 |  | 13.82 | 12.69 | 15.55 |
|  | **MdSeh1b** | MD14G1122800 | 2.95 | 4.72 | 6.91 | 7.61 | 7.29 | 7.60 | 4.91 | 5.12 | 6.52 |  | 2.51 | 2.14 | 2.51 |  | 0.00 | 2.71 | 0.61 |  | 9.70 | 9.80 | 12.28 |  | 7.72 | 10.06 | 8.45 |  | 5.92 | 6.71 | 5.96 |
| **Others subcomplex** | **MdNup50a** | MD09G1214400 | 28.68 | 29.30 | 27.40 | 29.14 | 30.89 | 31.10 | 28.57 | 29.19 | 30.21 |  | 11.51 | 12.06 | 13.47 |  | 13.20 | 11.18 | 14.48 |  | 24.24 | 24.93 | 22.14 |  | 32.26 | 29.69 | 27.13 |  | 26.19 | 25.72 | 24.65 |
|  | **MdNup50b** | MD17G1196800 | 34.92 | 31.75 | 34.39 | 35.84 | 37.38 | 36.69 | 33.78 | 37.20 | 34.68 |  | 21.14 | 23.41 | 21.73 |  | 20.77 | 20.01 | 22.05 |  | 31.17 | 29.94 | 28.95 |  | 36.48 | 32.02 | 27.73 |  | 22.23 | 25.33 | 25.04 |
|  | **MdNup88** | MD01G1152200 | 5.24 | 5.52 | 5.14 | 5.38 | 5.17 | 5.32 | 5.22 | 5.29 | 5.09 |  | 6.33 | 6.15 | 7.09 |  | 2.24 | 3.46 | 2.71 |  | 8.57 | 9.26 | 8.25 |  | 6.65 | 5.76 | 6.67 |  | 9.16 | 9.77 | 9.08 |
|  | **MdNup98a** | MD06G1126300 | 27.26 | 29.09 | 27.74 | 37.24 | 35.25 | 36.57 | 31.42 | 31.52 | 30.78 |  | 19.94 | 19.37 | 20.18 |  | 14.93 | 16.36 | 15.91 |  | 18.64 | 19.01 | 17.36 |  | 20.39 | 15.87 | 20.61 |  | 22.81 | 23.38 | 24.14 |
|  | **MdNup98b** | MD14G1142000 | 16.27 | 16.94 | 15.45 | 19.70 | 17.71 | 20.62 | 17.57 | 18.60 | 17.67 |  | 14.71 | 15.02 | 15.24 |  | 13.27 | 14.33 | 13.68 |  | 25.36 | 23.67 | 21.74 |  | 28.63 | 23.75 | 27.38 |  | 25.08 | 26.74 | 27.28 |
|  | **MdNup136a** | MD02G1257800 | 9.84 | 9.42 | 8.76 | 10.92 | 10.82 | 11.35 | 10.65 | 11.17 | 10.35 |  | 8.20 | 8.04 | 7.53 |  | 5.72 | 5.70 | 5.33 |  | 6.02 | 6.45 | 6.27 |  | 7.75 | 5.94 | 6.66 |  | 8.34 | 10.16 | 9.77 |
|  | **MdNup136b** | MD07G1063100 | 11.56 | 11.72 | 10.71 | 16.52 | 13.84 | 15.39 | 14.23 | 15.00 | 13.34 |  | 7.58 | 6.72 | 7.29 |  | 0.00 | 1.48 | 0.00 |  | 7.92 | 8.95 | 6.07 |  | 7.16 | 6.73 | 6.17 |  | 13.27 | 12.79 | 11.34 |
|  | **MdNDC1a** | MD05G1278400 | 9.39 | 9.66 | 11.20 | 10.37 | 10.68 | 10.06 | 10.15 | 10.20 | 10.82 |  | 10.06 | 10.00 | 9.95 |  | 10.40 | 9.83 | 9.36 |  | 13.16 | 13.71 | 14.13 |  | 14.02 | 12.19 | 12.88 |  | 10.90 | 10.59 | 11.85 |
|  | **MdNDC1b** | MD10G1257000 | 2.06 | 2.17 | 2.36 | 2.42 | 2.38 | 2.50 | 2.49 | 2.99 | 2.19 |  | 1.82 | 1.34 | 1.54 |  | 0.67 | 0.96 | 0.87 |  | 2.72 | 2.77 | 2.68 |  | 2.54 | 2.83 | 1.99 |  | 2.20 | 1.53 | 1.98 |
|  | **MdTpr/NUA** | MD05G1240600 | 18.00 | 19.40 | 17.09 | 18.86 | 18.44 | 20.75 | 19.98 | 18.68 | 17.76 |  | 12.52 | 12.56 | 12.58 |  | 7.06 | 5.90 | 7.41 |  | 11.76 | 11.20 | 11.45 |  | 9.23 | 8.55 | 9.43 |  | 18.96 | 20.07 | 19.67 |
|  | **MdCGI** | MD04G1000600 | 15.26 | 15.61 | 15.56 | 18.29 | 16.06 | 18.70 | 16.07 | 16.30 | 16.63 |  | 12.74 | 12.24 | 13.27 |  | 5.08 | 6.95 | 5.80 |  | 12.38 | 11.80 | 13.98 |  | 13.66 | 13.35 | 13.44 |  | 13.72 | 14.37 | 14.62 |
|  | **MdRAE1a** | MD08G1221600 | 19.08 | 17.14 | 15.84 | 17.71 | 16.53 | 15.51 | 14.66 | 17.57 | 18.78 |  | 22.41 | 22.95 | 23.55 |  | 26.59 | 24.54 | 26.21 |  | 30.25 | 32.27 | 28.46 |  | 46.43 | 39.47 | 38.34 |  | 30.40 | 27.88 | 24.99 |
|  | **MdRAE1b** | MD15G1412500 | 5.48 | 6.14 | 4.78 | 5.87 | 4.54 | 5.92 | 7.05 | 7.16 | 5.59 |  | 1.41 | 2.19 | 1.68 |  | 6.16 | 6.10 | 5.80 |  | 7.05 | 6.17 | 6.74 |  | 3.63 | 4.39 | 3.58 |  | 5.31 | 4.39 | 4.68 |
|  | **MdALADIN** | MD12G1112000 | 10.73 | 12.88 | 12.94 | 12.87 | 14.53 | 14.19 | 15.19 | 11.95 | 13.92 |  | 12.54 | 14.05 | 11.47 |  | 0.00 | 0.00 | 0.07 |  | 12.31 | 11.53 | 14.54 |  | 9.80 | 10.79 | 9.63 |  | 9.14 | 9.67 | 10.64 |
|  | **MdGP210** | MD17G1026700 | 8.21 | 8.18 | 6.84 | 8.20 | 8.14 | 8.69 | 9.37 | 10.15 | 9.55 |  | 7.37 | 7.98 | 7.85 |  | 1.69 | 1.49 | 2.60 |  | 9.11 | 7.79 | 7.70 |  | 6.98 | 6.47 | 5.79 |  | 7.29 | 7.84 | 6.03 |
|  | **MdHOS1** | MD04G1060900 | 9.66 | 12.19 | 9.85 | 12.53 | 11.06 | 12.70 | 13.19 | 12.29 | 12.97 |  | 17.53 | 16.05 | 18.48 |  | 0.00 | 0.00 | 0.00 |  | 12.39 | 12.13 | 13.71 |  | 8.66 | 8.02 | 8.67 |  | 16.83 | 16.51 | 14.57 |
|  | **MdGLE1** | MD13G1104500 | 22.07 | 21.93 | 22.27 | 22.11 | 21.64 | 24.65 | 22.17 | 22.33 | 22.44 |  | 13.49 | 15.97 | 15.92 |  | 7.48 | 7.31 | 7.99 |  | 15.06 | 16.08 | 16.15 |  | 17.01 | 15.32 | 14.31 |  | 12.28 | 10.23 | 11.06 |

Note: The full names of the different abbreviations are as follows, ‘Nagafu No.2’ long branches flower buds (FLB), ‘Nagafu No.2’ short branches flower buds (FSB), ‘Nagafu No.2’ axillary buds (FYB), ‘Qinguan’ axillary buds (QYB), ‘Nagafu No.2’ fruit (FR), ‘Yanfu No.3’ stem tip (YF3J), ‘Yanfu No.6’ stem tip (YF6J), and ‘M9-T337’ root (T337R). Each number after the abbreviation represents a biological repetition. The same below.

**Table S2. Expression of *MdHSFs* in different tissues of several apple varieties**

|  |  | **‘Nagafu No.2’** | | | | | | | | |  | **‘Qinguan’** | | |  | **‘Nagafu No.2’** | | |  | **‘Yanfu No.3’** | | |  | **‘Yanfu No.6’** | | |  | **‘M9-T337’** | | |
| --- | --- | --- | --- | --- | --- | --- | --- | --- | --- | --- | --- | --- | --- | --- | --- | --- | --- | --- | --- | --- | --- | --- | --- | --- | --- | --- | --- | --- | --- | --- |
| **Gene** | **Gene ID** | **FLB1** | **FLB2** | **FLB3** | **FSB1** | **FSB2** | **FSB3** | **FYB1** | **FYB2** | **FYB3** |  | **QYB1** | **QYB2** | **QYB3** |  | **FR1** | **FR2** | **FR3** |  | **YF3J1** | **YF3J2** | **YF3J3** |  | **YF6J1** | **YF6J2** | **YF6J3** |  | **T337R1** | **T337R2** | **T337R3** |
| **MdHSFA9b** | MD15G1209400 | 7.33 | 7.97 | 7.57 | 8.9 | 9.03 | 7.49 | 8.19 | 8.9 | 7.7 |  | 0.66 | 1.25 | 1.17 |  | 0.46 | 0.37 | 0 |  | 0.61 | 0.53 | 0.22 |  | 0.84 | 0.51 | 0.38 |  | 2.15 | 2.84 | 3.14 |
| **MdHSFA9a** | MD02G1082000 | 10.27 | 7.57 | 8.1 | 8.94 | 8.1 | 8.18 | 7.2 | 7.66 | 7.2 |  | 11.55 | 11.2 | 10.74 |  | 0.24 | 0.07 | 0.14 |  | 3.32 | 2.61 | 2.6 |  | 1.77 | 2.35 | 0.71 |  | 0.47 | 0.26 | 0.29 |
| **MdHSFA1d** | MD16G1271200 | 8.21 | 6.14 | 7.23 | 6.67 | 7.47 | 7.4 | 8.19 | 8.16 | 8.75 |  | 5.43 | 5.86 | 5.42 |  | 2.05 | 2.3 | 2.52 |  | 8.65 | 8.38 | 8.24 |  | 9.12 | 7.85 | 7.73 |  | 8.78 | 9.56 | 8.7 |
| **MdHSFA1a** | MD06G1037900 | 8.86 | 8.05 | 8.33 | 10.02 | 8.14 | 10.01 | 9.75 | 9.16 | 9.33 |  | 13.64 | 13.45 | 12.68 |  | 0 | 0 | 0 |  | 10.4 | 11.12 | 13.84 |  | 22.18 | 19.95 | 20.12 |  | 14.98 | 14.09 | 13.31 |
| **MdHSFA1e** | MD05G1213800 | 10.79 | 11.47 | 11.23 | 9.75 | 10.59 | 11.79 | 10.54 | 12.22 | 11.6 |  | 7.05 | 7.43 | 7.98 |  | 1.66 | 1.59 | 1.53 |  | 4.78 | 4.75 | 5.44 |  | 4 | 3.99 | 3.89 |  | 4.48 | 6.28 | 6.04 |
| **MdHSFA1b** | MD10G1197700 | 14.12 | 12.87 | 14.52 | 13.74 | 13.08 | 14.61 | 15.63 | 15.46 | 15.24 |  | 6.68 | 7.07 | 6.09 |  | 2.39 | 1.89 | 2.22 |  | 3.86 | 2.92 | 3.5 |  | 3.43 | 3.1 | 2.86 |  | 6.7 | 7.78 | 6.88 |
| **MdHSFB4b** | MD15G1080700 | 2.53 | 1.12 | 1.27 | 1.35 | 1.62 | 1.88 | 0.96 | 1.15 | 1.19 |  | 2.24 | 2.81 | 2.28 |  | 0 | 0.03 | 0 |  | 12.52 | 9.17 | 14.78 |  | 3.44 | 4.18 | 2.73 |  | 5.54 | 5.89 | 6.03 |
| **MdHSFB4a** | MD08G1098200 | 0.95 | 0.56 | 0.87 | 0.72 | 0.65 | 0.95 | 0.55 | 0.46 | 0.36 |  | 0.28 | 0.81 | 0.82 |  | 0 | 0 | 0 |  | 2.84 | 2.02 | 3.37 |  | 2.45 | 1.35 | 1.15 |  | 6.96 | 8.09 | 7.03 |
| **MdHSFA8b** | MD16G1016700 | 12.41 | 12.7 | 12.57 | 12.61 | 13.79 | 13 | 11.74 | 13.61 | 15.21 |  | 30.07 | 31.32 | 30.15 |  | 4.65 | 4.92 | 4.56 |  | 20.75 | 24.33 | 18.33 |  | 20.25 | 15.22 | 17.93 |  | 10.63 | 9.94 | 8.95 |
| **MdHSFA8a** | MD13G1018800 | 0.93 | 1.37 | 1.1 | 0.84 | 1.24 | 1.3 | 1.38 | 1 | 1.19 |  | 8.87 | 8.83 | 8.77 |  | 0 | 0 | 0 |  | 9.38 | 10.53 | 11.31 |  | 3.79 | 3.14 | 4.33 |  | 3.75 | 3.07 | 3.15 |
| **MdHSFA2a** | MD08G1064100 | 3.68 | 4.41 | 4.58 | 5.8 | 4.37 | 6.41 | 6.65 | 7.43 | 6.63 |  | 20.92 | 20.96 | 20.59 |  | 0 | 0 | 0 |  | 5.92 | 8.07 | 2.69 |  | 8.93 | 20.42 | 11.26 |  | 0.36 | 0.07 | 0.35 |
| **MdHSFA2b** | MD15G1057700 | 30.46 | 32.36 | 29.64 | 38.76 | 38.65 | 41.64 | 38.8 | 34.15 | 35.52 |  | 277.52 | 278.88 | 286.54 |  | 0.79 | 0.88 | 0.85 |  | 245.14 | 271.68 | 144.21 |  | 1070.79 | 1313.29 | 910.96 |  | 13.2 | 12.15 | 12.2 |
| **MdHSFB3** | MD12G1079300 | 0.25 | 0.69 | 0.4 | 0 | 0.16 | 0.15 | 0.48 | 0.37 | 0.35 |  | 1.81 | 1.96 | 1.8 |  | 0 | 0 | 0 |  | 0.68 | 1.55 | 0.31 |  | 2.74 | 1.36 | 1.01 |  | 27.81 | 27.46 | 26.8 |
| **MdHSFA6b** | MD16G1199800 | 0 | 0.13 | 0 | 0 | 0 | 0 | 0 | 0.07 | 0 |  | 0.55 | 1.25 | 0.63 |  | 0 | 0 | 0 |  | 0.36 | 0.16 | 0 |  | 0 | 0.05 | 0 |  | 3.45 | 3.6 | 3.31 |
| **MdHSFA6a** | MD13G1199700 | 0 | 0 | 0 | 0 | 0 | 0 | 0 | 0 | 0 |  | 0 | 0 | 0 |  | 0 | 0 | 0 |  | 0.05 | 0 | 0 |  | 0 | 0 | 0 |  | 0.21 | 0.58 | 0.38 |
| **MdHSFC1a** | MD02G1046900 | 35.78 | 32.6 | 38.12 | 33.67 | 40.12 | 33.25 | 36.28 | 32.73 | 36.76 |  | 1.03 | 1.06 | 0.54 |  | 0.15 | 0.45 | 0.33 |  | 0.38 | 0.24 | 0.21 |  | 0.81 | 0.3 | 0.45 |  | 2.6 | 2.72 | 2.99 |
| **MdHSFC1b** | MD15G1185400 | 213.4 | 214.37 | 222.53 | 144.33 | 144.32 | 144.15 | 160.56 | 158.46 | 159.27 |  | 16.54 | 14.8 | 16.55 |  | 5.78 | 4.53 | 5.39 |  | 26.69 | 26.75 | 28.8 |  | 4.45 | 4.25 | 10.1 |  | 6.08 | 6.7 | 5.22 |
| **MdHSFA7a** | MD03G1258300 | 0.79 | 0.64 | 1.24 | 1.26 | 0.98 | 0.87 | 0.82 | 0.87 | 1.17 |  | 4.08 | 4.64 | 4.34 |  | 0.39 | 0.51 | 0.48 |  | 10.43 | 9.26 | 6.28 |  | 59.49 | 76.01 | 44.19 |  | 18.14 | 19.59 | 17.98 |
| **MdHSFA7b** | MD11G1278900 | 0.18 | 0.33 | 0.1 | 0.06 | 0.09 | 0.16 | 0.17 | 0.11 | 0.05 |  | 1.38 | 1.45 | 1.45 |  | 0 | 0 | 0 |  | 5.71 | 7.42 | 8.29 |  | 12.76 | 11.2 | 9.71 |  | 5.77 | 5.24 | 4.79 |
| **MdHSFB2b** | MD04G1064700 | 14.61 | 13.15 | 13.22 | 15 | 12.72 | 13.91 | 12.57 | 10.14 | 10.57 |  | 58.76 | 54.05 | 56.15 |  | 32.29 | 31.88 | 35.58 |  | 47.44 | 47.8 | 47.73 |  | 113.57 | 95.71 | 105.18 |  | 5.08 | 5.34 | 5.82 |
| **MdHSFB2c** | MD07G1266500 | 8.13 | 8.38 | 9.09 | 6.33 | 5.89 | 6.12 | 7.77 | 9.39 | 8.67 |  | 82.92 | 84.12 | 84.4 |  | 0.73 | 0.5 | 0.7 |  | 55.62 | 52.55 | 60.47 |  | 118.39 | 104.62 | 92.83 |  | 2.36 | 1.66 | 1.89 |
| **MdHSFB2a** | MD01G1198700 | 9.6 | 9.55 | 10.47 | 10.82 | 10.59 | 8.3 | 10.56 | 9.57 | 12.37 |  | 9.88 | 9.85 | 11.57 |  | 0 | 0.11 | 0.05 |  | 10.33 | 10.6 | 8.22 |  | 51.55 | 44.51 | 34.33 |  | 0.23 | 0.29 | 0.25 |
| **MdHSFA5a** | MD00G1118600 | 8.51 | 9.54 | 8.91 | 8.17 | 7.96 | 9.01 | 9.07 | 9.39 | 9.51 |  | 14.37 | 14 | 15.08 |  | 1.42 | 1.32 | 1.51 |  | 6.9 | 6.99 | 6.67 |  | 18.75 | 15.18 | 15.42 |  | 8.08 | 8.75 | 9.17 |
| **MdHSFA5b** | MD04G1017400 | 8.84 | 10.05 | 10.38 | 7.53 | 8.49 | 7.1 | 6.9 | 7.31 | 8.66 |  | 12.36 | 12.34 | 13.12 |  | 0 | 1.48 | 1.79 |  | 7.67 | 7.47 | 7.11 |  | 24.54 | 17.01 | 19.61 |  | 9.06 | 10.44 | 11.17 |
| **MdHSFA4b** | MD10G1274400 | 8.52 | 6.91 | 7.76 | 7.86 | 8.82 | 8.47 | 6.61 | 8.19 | 7.79 |  | 4.27 | 3.47 | 4.34 |  | 2.92 | 2.89 | 2.48 |  | 2.97 | 2.64 | 3.3 |  | 1.41 | 1.59 | 1.57 |  | 4.45 | 3.49 | 3.32 |
| **MdHSFA4a** | MD00G1007800 | 10.74 | 10.46 | 10.76 | 10.39 | 11.17 | 10.68 | 9.05 | 9.91 | 9.85 |  | 26.39 | 28.04 | 27.88 |  | 3.96 | 2.88 | 3.98 |  | 12.16 | 14.72 | 14.22 |  | 9.2 | 7.09 | 10.51 |  | 18.85 | 19.63 | 19.29 |
| **MdHSFB1a** | MD02G1171800 | 7.41 | 7.48 | 7.25 | 3.54 | 4.78 | 4.23 | 5.36 | 5.56 | 5.05 |  | 79.39 | 79.04 | 79.75 |  | 0.42 | 0.35 | 0.52 |  | 9.57 | 8.44 | 5.81 |  | 15.73 | 17.84 | 10.86 |  | 74.02 | 73.4 | 84.21 |
| **MdHSFB1b** | MD15G1283700 | 45.95 | 41.31 | 45.29 | 21.06 | 24.83 | 20.53 | 36.77 | 32.46 | 34.44 |  | 51.7 | 49.68 | 51.16 |  | 78.57 | 70.63 | 73.83 |  | 2.19 | 4.84 | 1.57 |  | 5.43 | 2.55 | 3.47 |  | 103.93 | 104.08 | 117.66 |
| **MdHSFA3** | MD14G1015900 | 3.84 | 2.9 | 2.95 | 2.71 | 3.44 | 2.86 | 3.23 | 2.77 | 3.76 |  | 7.13 | 8.23 | 8.28 |  | 0.55 | 0.57 | 0.4 |  | 1.4 | 1.5 | 0.97 |  | 78.01 | 69.57 | 114.86 |  | 7.41 | 7.24 | 5.67 |

**Table S3. MdNup62 yeast double-hybridization screening results**

| **NCBI GenBank ID** | **Annotation** |
| --- | --- |
| LOC103436919 | Malus x domestica heat shock factor protein HSF8-like |
| LOC103440772 | Malus x domestica heat shock factor protein HSF30-like |
| NM_001294365.1 | Malus x domestica Heat shock transcription factor 1 (HSF1) |
| LOC103453152 | Malus x domestica heat stress transcription factor A-1-like (HSFA1E) |
| LOC103403678 | Malus x domestica heat stress transcription factor A-1d |
| LOC103425010 | Malus x domestica heat stress transcription factor A-2-like |
| LOC103440054 | Malus x domestica myb-like protein V |
| LOC103404780 | Malus x domestica transcription factor MYC2-like |
| LOC103439016 | Malus x domestica nuclear pore complex protein NUP62 |
| LOC103422860 | Malus x domestica GATA transcription factor 11-like |
| LOC103455855 | Malus x domestica squalene monooxygenase |
| LOC103455002 | Malus x domestica HVA22-like protein in response to cold stress, salt stress, ABA treatment or dehydration. |
| LOC103428433 | Malus x domestica MATH domain and coiled-coil domain-containing protein At3g58210-like |
| LOC103450707 | Malus x domestica patellin-6-like |
| LOC103433685 | Malus x domestica ferritin-4, chloroplastic-like |
| LOC103448276 | Malus x domestica B3 domain-containing protein Os01g0234100-like |
| LOC103419975 | Malus x domestica A BAK1-interacting receptor-like kinase named BIR1. |
| LOC103438249 | Malus x domestica acetyl-CoA carboxylase 1-like |
| LOC103408896 | Malus x domestica calcyclin-binding protein-like |
| NM_001294052.1 | Malus domestica transcription factor MYB21 (MYB7) |
| LOC103409074 | Malus x domestica TMV resistance protein N-like |
| LOC103454691 | Malus x domestica protein PHR1-LIKE 1-like（myb-like HTH transcriptional regulator family protein） |
| LOC103438971 | Malus x domestica E3 ubiquitin-protein ligase BRE1-like 1 |
| LOC103414336 | Malus x domestica nuclear pore complex protein NUP54-like |
| LOC103433332 | Malus x domestica probable LRR receptor-like serine/threonine-protein kinase |
| LOC103409229 | Malus x domestica MATH domain and coiled-coil domain-containing protein At3g58270-like |
| LOC103438633 | Malus x domestica MATH domain and coiled-coil domain-containing protein At3g44800-like |
| LOC103424391 | Malus x domestica flagellar attachment zone protein 1-like |
| LOC103452265 | Malus x domestica CDP-diacylglycerol--serine O-phosphatidyltransferase 1 |
| LOC103452902 | Malus x domestica kinesin-13A-like |
| LOC103400469 | Malus x domestica U1 small nuclear ribonucleoprotein 70 kDa |
| LOC103451871 | Malus x domestica uncharacterized LOC103451871 AtPTST2 |
| LOC110601849 | Manihot esculenta putative leucine-rich repeat-containing protein DDB_G0290503 |
| LOC103448737 | Malus x domestica basic leucine zipper 34-like |
| LOC103447177 | Malus x domestica WEB family protein At5g55860 |
| LOC103442489 | Malus x domestica protein CROWDED NUCLEI 1 |
| LOC108171634 | Malus x domestica uncharacterized LOC108171634 |
| LOC108173799 | Malus x domestica 50S ribosomal protein L27-like |
| LOC103452600 | Malus x domestica 1,4-dihydroxy-2-naphthoyl-CoA thioesterase 1 |
| LOC103445122 | Malus x domestica kinesin-like protein KIN-7D, mitochondrial |
| LOC103444128 | Malus x domestica uncharacterized |
| LOC103442480 | Malus x domestica protein FLX-like 2 |
| LOC103446377 | Malus x domestica dnaJ homolog subfamily B member 1-like |
| LOC103444557 | Malus x domestica uncharacterized |
| LOC103447261 | Malus x domestica protein NETWORKED 4B |
| LOC103400142 | Malus x domestica uncharacterized |
| LOC103432650 | Malus x domestica uncharacterized |
| LOC103428182 | Malus x domestica glucosamine 6-phosphate N-acetyltransferase-like |
| LOC103403542 | Malus x domestica aldehyde dehydrogenase 22A1 |
| LOC103424142 | Malus x domestica RWD domain-containing protein 1-like |
| LOC103432529 | Malus x domestica uncharacterized |
| LOC103423350 | Malus x domestica UDP-glycosyltransferase 86A1-like |
| LOC103410066 | Malus x domestica acyl-CoA-binding domain-containing protein 4-like |
| LOC103432980 | Malus x domestica deSI-like protein At4g17486 |
| LOC103443038 | Malus x domestica vacuolar protein sorting-associated protein 53 A-like |
| LOC103404478 | Malus x domestica chorismate mutase 1, chloroplastic |
| LOC103411108 | Malus x domestica alpha-mannosidase-like |
| LOC103412154 | Malus x domestica 40S ribosomal protein SA-like |
| LOC103448513 | Malus x domestica U1 small nuclear ribonucleoprotein 70 kDa-like |
| LOC103423604 | Malus x domestica zinc finger CCCH domain-containing protein ZFN-like |
| LOC103453633 | Malus x domestica subtilisin-like protease SBT1.4 |

**Table S4. Primers used for qRT-PCR**

| **Gene name** | **Gene ID** | **Primer sequences** |
| --- | --- | --- |
| AtFT-F | AT1G65480 | GGTGACTAATGGCTTGGAT |
| AtFT-R | AT1G65480 | GGACTTGGAACATCTGGAT |
| AtSOC1-F | AT2G45660 | GCAACAAGCAGACAAGTG |
| AtSOC1-R | AT2G45660 | CTTAGTATGCCTCAGATAACG |
| AtLFY-F | AT5G61850 | CCGTGAGTTCCTTCTTCAG |
| AtLFY-R | AT5G61850 | CTTCTTCGTCTAGGCAGTG |
| AtAP1-F | AT1G69120 | GGCAATGAGGAGGAATGAT |
| AtAP1-R | AT1G69120 | GAGCCTAGCCACTATTTATATG |
| AtActin-F | AT2G37620 | GCGATTCCGTTGTCCTGAGGTTC |
| AtActin-R | AT2G37620 | TTCCACCACTGAGCACAATGTTACC |
| AtHSP101-F | AT1G74310 | AAGATGGTTGTGCGTGAG |
| AtHSP101-R | AT1G74310 | CTGACTTCTTGCCTGTTGA |
| AtHSP22-ER-F | AT4G10250 | ACCAGAGAATTGAGACTAACC |
| AtHSP22-ER-R | AT4G10250 | GCTCCTATGAAGAAGATGCT |
| AtHSP70T-2-F | AT2G32120 | ACAGCAGCAGATGACTAC |
| AtHSP70T-2-R | AT2G32120 | TAGCAGTAACCGCAACAT |
| AtHSP21-F | AT4G27670 | GGCTTCTACACTCTCATTTGC |
| AtHSP21-R | AT4G27670 | GGTCTTGAGCCCTGATCC |
| SlActin-F | Solyc11g005330 | GTCCTCTTCCAGCCATCCATGA |
| SlActin-R | Solyc11g005330 | ACCACTGAGCACAATGTTACCG |
| SlHSP70T-2-F | Solyc09g075950 | TAAGATTGATGCCTGAACCA |
| SlHSP70T-2-R | Solyc09g075950 | ACCTGCTGTAGCTGTTATG |
| SlHSP101-F | Solyc03g115230 | GCCATCCAAGAAGTAGAATC |
| SlHSP101-R | Solyc03g115230 | TCTATCAGCAAGACCAATCA |
| SlHSP22.0-ER-F | Solyc11g020330 | CGCAGATGGGTCATCACTTG |
| SlHSP22.0-ER-R | Solyc11g020330 | ACTCTCGCGAGCAGAGTG |
| SlHSP21-F | Solyc03g082420 | GACACTATGGACAGATTATTCG |
| SlHSP21-R | Solyc03g082420 | TACATCTTCCTTGGACAGTC |
| MdActin-F | MD04G1127400 | CAACTCATCCGAACCTCAAACC |
| MdActin-R | MD04G1127400 | CGCTGTCCGCCATCTTCTACT |
| MdHSFA9b-F | MD15G1209400 | AAGAAGAAGTGACCGAACTGAA |
| MdHSFA9b-R | MD15G1209400 | CATCCTCCACAACCACAACAT |
| MdHSFA1d-F | MD16G1271200 | ACTTCTTGATTGGTGATGGTTC |
| MdHSFA1d-R | MD16G1271200 | CCGCAGAGGAGATTGTGTT |
| MdNUP62-F | MD07G1110700 | GGCTTCCTCCGCTTCACAATCC |
| MdNUP62-R | MD07G1110700 | CGGTGCTAGGAGCCTGAGAAGT |

**Table S5. Primers used for plasmid construction**

| **Gene name** | **Gene ID** | **Primer sequences** |
| --- | --- | --- |
| MdNup62-F | MD07G1110700 | CCTCTCAATCACATAGAGCAGCAG |
| MdNup62-R | MD07G1110700 | GGAATTGAAGGTGGTTTACAATTCC |
| MdNUP62508-613-pGBKT7-F | MD07G1110700 | ATGGCCATGGAGGCCGAATTCGTCAACACTACAGTATC |
| MdNUP62508-613-pGBKT7-R | MD07G1110700 | CCGCTGCAGGTCGACGGATCCCTTGTATATACGCTCAG |
| MdNUP62-NLUC-F | MD07G1110700 | GCTCTAGAATGTCGGGATTTTCATCCGG |
| MdNUP62-NLUC-R | MD07G1110700 | CGCGGATCCCGACATCCAGTATTTTGGAGC |
| MdHSFA9a-pGADT7-F | MD02G1082000 | GCCATGGAGGCCAGTGAATTCATGGTGGATCCTGACGTT |
| MdHSFA9a-pGADT7-R | MD02G1082000 | CAGCTCGAGCTCGATGGATCCGCCAATGCAGCCAGCTTG |
| MdHSFA9b-pGADT7-F | MD15G1209400 | GCCATGGAGGCCAGTGAATTCATGGTGGTTCCCGACGG |
| MdHSFA9b-pGADT7-R | MD15G1209400 | CAGCTCGAGCTCGATGGATCCAGGCACTGAGCCAATGCA |
| MdHSFA1a-pGADT7-F | MD06G1037900 | GCCATGGAGGCCAGTGAATTCATGGGGGGCGCTGATAAC |
| MdHSFA1a-pGADT7-R | MD06G1037900 | CAGCTCGAGCTCGATGGATCCTACCCCTTTGGTATCTGA |
| MdHSFA1b-pGADT7-F | MD10G1197700 | GCCATGGAGGCCAGTGAATTCATGCAAACAACCATGGAG |
| MdHSFA1b-pGADT7-R | MD10G1197700 | CAGCTCGAGCTCGATGGATCCACCAATTCTGCTCCCTGAT |
| MdHSFA1d-pGADT7-F | MD16G1271200 | GCCATGGAGGCCAGTGAATTCATGGAGGGTGCTAATAAC |
| MdHSFA1d-pGADT7-R | MD16G1271200 | CAGCTCGAGCTCGATGGATCCCACCCCTTTGGTCTCTGA |
| MdHSFA1e-pGADT7-F | MD05G1213800 | GCCATGGAGGCCAGTGAATTCATGCAAACAACCATGGAG |
| MdHSFA1e-pGADT7-R | MD05G1213800 | CAGCTCGAGCTCGATGGATCCACCAATTCTGCTCCCTGA |
| MdNup62-PC2300-F | MD07G1110700 | ACGAGCTCGGTACCATGTCGGGATTTTCATCCGG |
| MdNup62-PC2300-R | MD07G1110700 | CATGGTGTCGACTCTAGACGACATCCAGTATTTTGGAGC |
| MdHSFA9b-PC2300-F | MD15G1209400 | ACGAGCTCGGTACCATGGTGGTTCCCGACGG |
| MdHSFA9b-PC2300-R | MD15G1209400 | CATGGTGTCGACTCTAGAAGGCACTGAGCCAATGCA |
| MdHSFA1d-PC2300-F | MD16G1271200 | ACGAGCTCGGTACCATGGAGGGTGCTAATAAC |
| MdHSFA1d-PC2300-R | MD16G1271200 | CATGGTGTCGACTCTAGACACCCCTTTGGTCTCTGA |
| MdNUP54-CLUC-F | MD16G1117500 | GCTCTAGAATGTTCGGAGCTCAATCTTCG |
| MdNUP54-CLUC-R | MD16G1117500 | CGCGGATCCGCTCCCGTTCTGTGTTGTT |
| MdHSFA9b-CLUC-F | MD15G1209400 | GATCGCCGTGTCTAGAATGGTGGTTCCCGACGG |
| MdHSFA9b-CLUC-R | MD15G1209400 | GCTTGATATCGAATTCAGGCACTGAGCCAATGCA |
| MdHSFA1d-CLUC-F | MD16G1271200 | GATCGCCGTGTCTAGAATGGAGGGTGCTAATAAC |
| MdHSFA1d-CLUC-R | MD16G1271200 | GCTTGATATCGAATTCCACCCCTTTGGTCTCTGA |
| MdHSFA9b-GST-F | MD15G1209400 | TGTTCCAGGGGCCCCTGGGATCCGTGGTTCCCGACGGTGG |
| MdHSFA9b-GST-R | MD15G1209400 | GCTCGAGTCGACCCGGGAATTCAGGCACTGAGCCAATGCA |
| MdNup62-HIS-F | MD07G1110700 | atgggtcgcggatccATGTCGGGATTTTCATCCGG |
| MdNup62-HIS-R | MD07G1110700 | gtggtgctcgagCGACATCCAGTATTTTGGAGC |

**
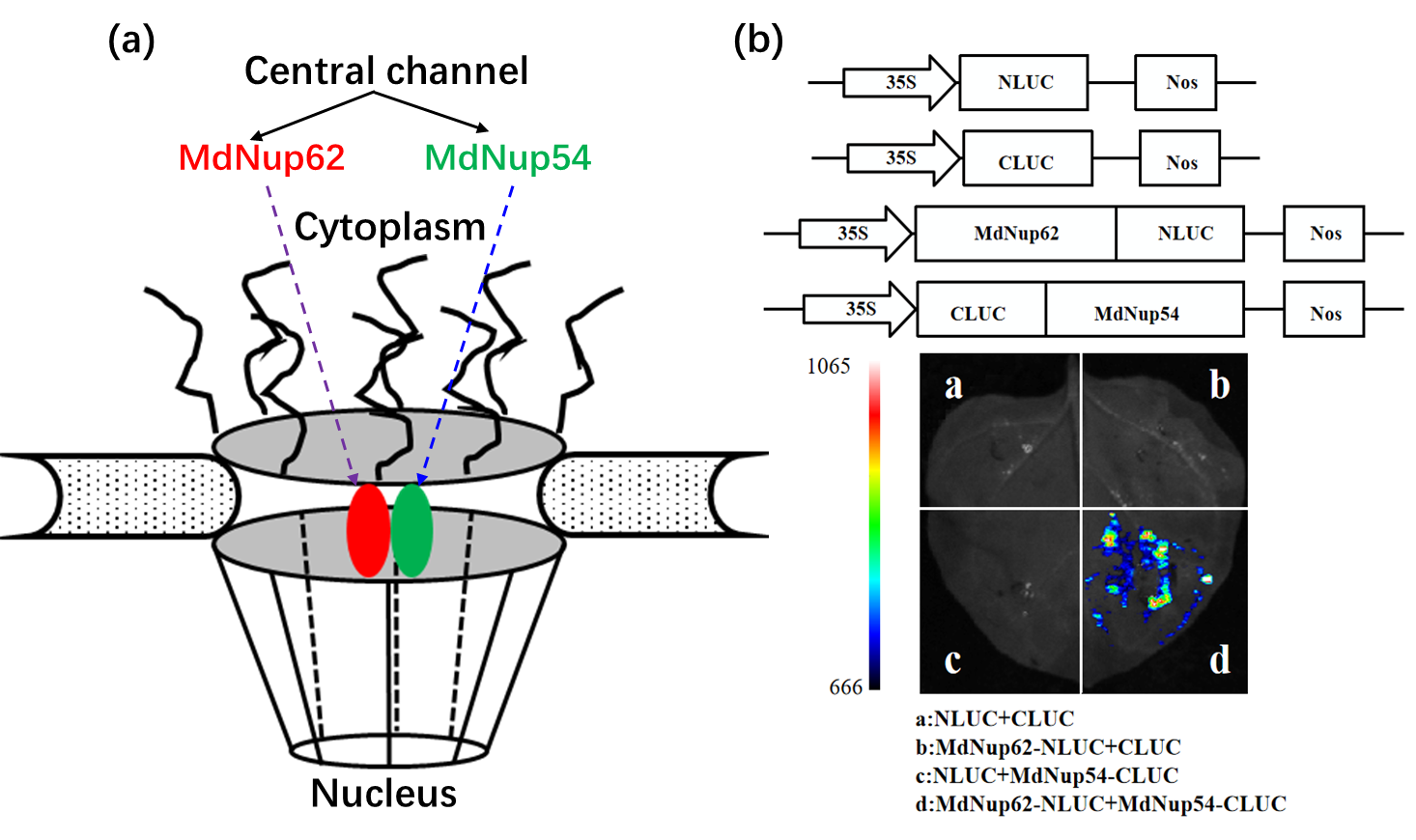
**

**Figure S1. Interactions between MdNup62 and MdNup54 in a luciferase (LUC) complementation experiment.**

(a) A schematic of the nuclear pore with the cytoplasmic side at the top and the nuclear basket at the bottom for Vertebrate (left) and Malus (right). MdNup62 interacts with MdNup54 forming the central NPC channel. (b) Interactions between MdNup62 and MdNup54 in a luciferase (LUC) complementation experiment, as well as in our previous study^18^. Empty NLUC and empty CLUC, *MdNup62*-NLUC plus empty CLUC, empty NLUC plus *MdNup54*-CLUC were used as controls.


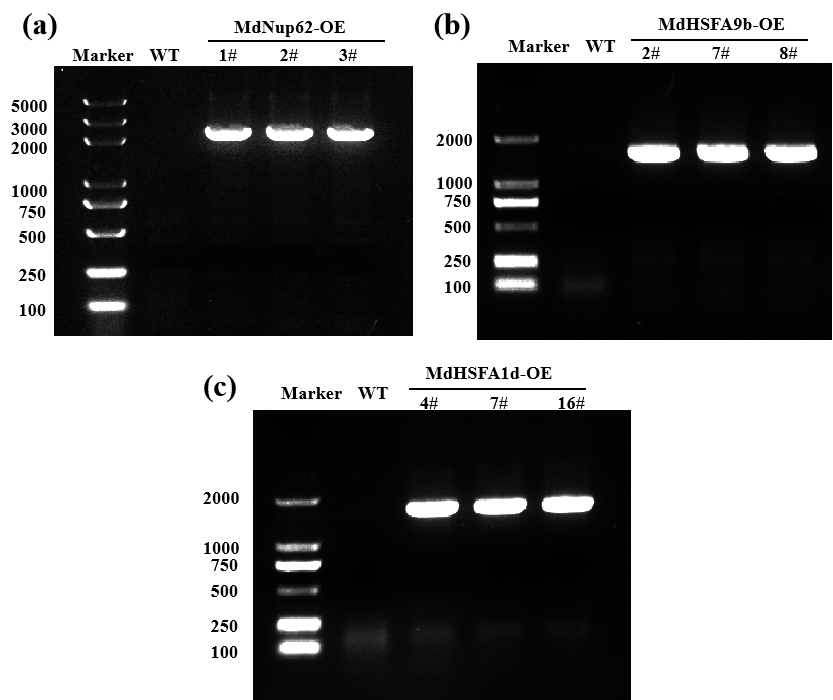


**Figure S2. Genomic PCR analyses of *MdNup62* (a), *MdHSFA9b* (b), and *MdHSFA1d* (c) in transgenic Arabidopsis lines**


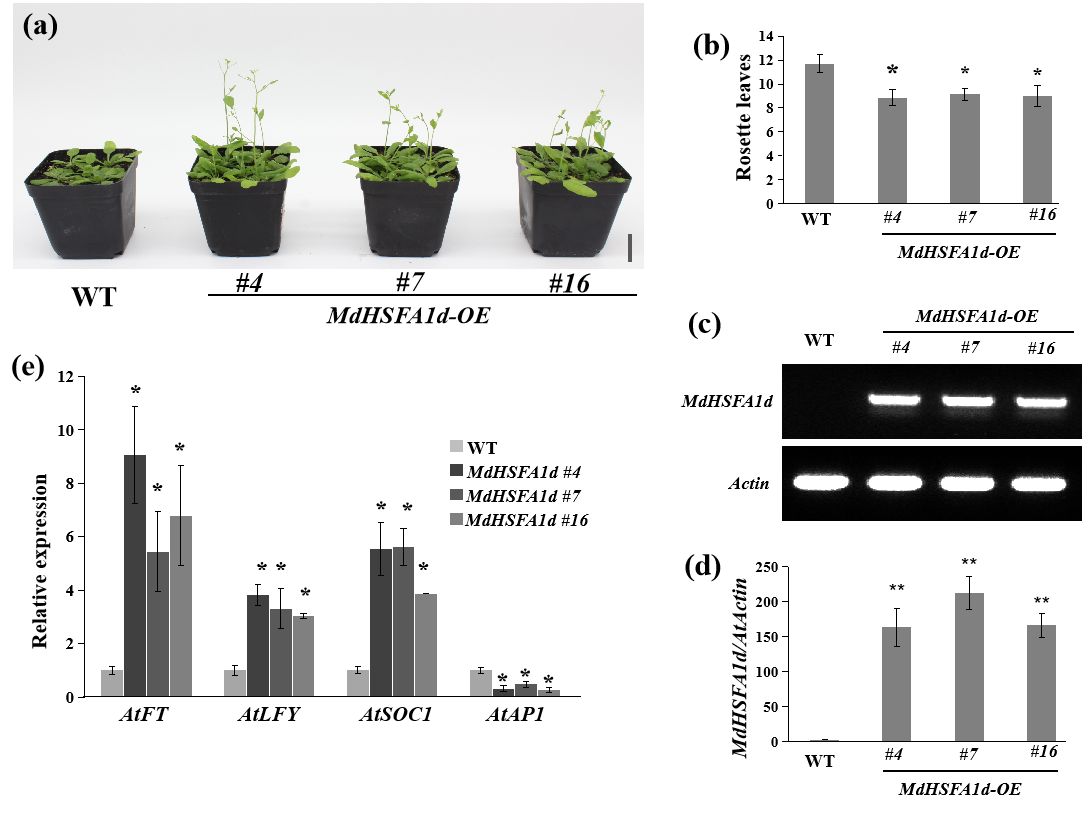


**Figure S3. *MdHSFA1d* promotes flowering in Arabidopsis.**

(a) Phenotype of the *MdHSFA1d*-overexpression Arabidopsis line for flowering time. Bar = 2 cm. (b) Statistical analysis of rosette leaves of *Arabidopsis thaliana* during bolting. (c) Semi-quantitative RT-PCR analysis of *MdHSFA1d* expression in Arabidopsis samples. (d) qRT-PCR analysis of *MdHSFA1d* expression in Arabidopsis samples. (e) Relative expression levels of flowering genes (*AtFT*, *AtLFY*, *AtSOC1*, and *AtAP1*) in WT and *MdHSFA1d*-overexpression lines.


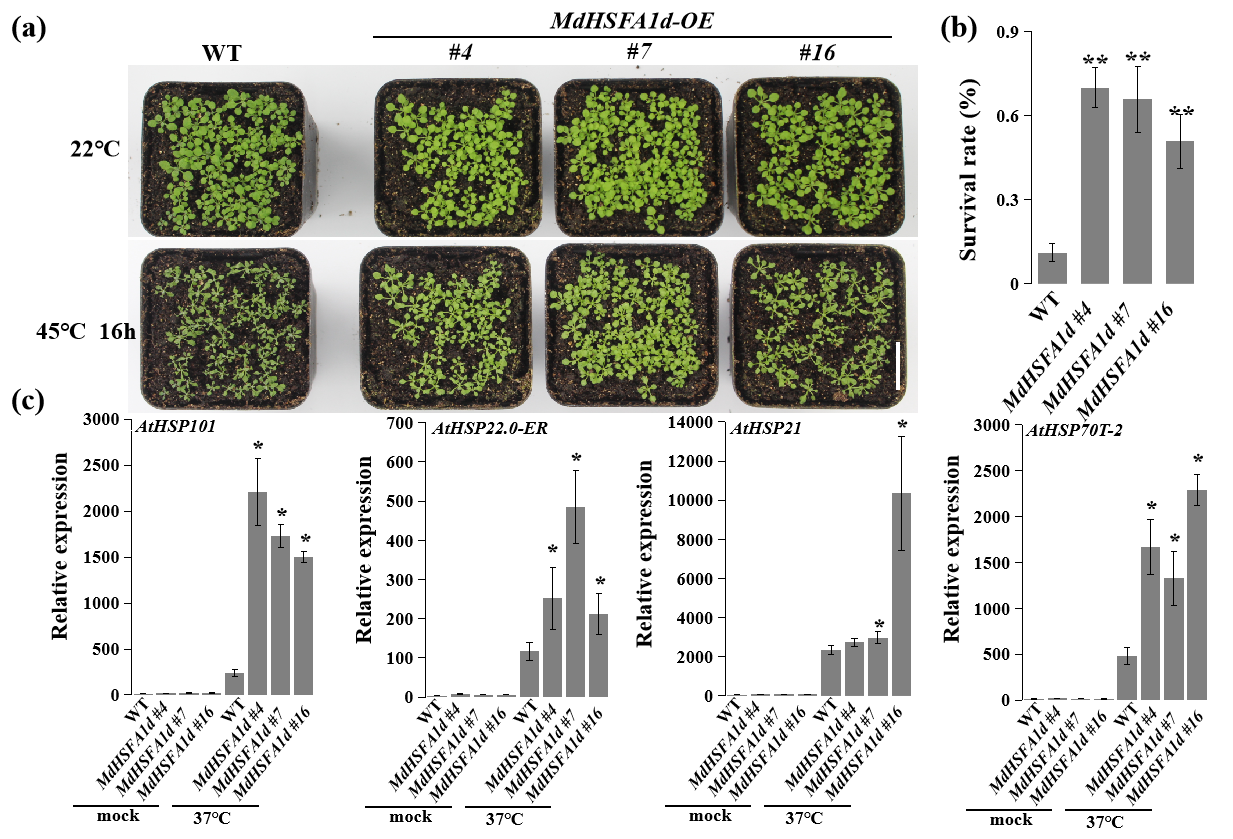


**Figure S4. *MdHSFA1d* enhanced high-temperature resistance in Arabidopsis.**

(a) Phenotype of the *MdHSFA1d*-overexpression Arabidopsis line for high-temperature resistance. Bar = 2 cm. (b) Survival rates of WT and *MdHSFA1d*-overexpression Arabidopsis lines after the high-temperature treatment. (c) Relative expression levels of high-temperature resistance-related genes (*AtHSP101*, *AtHSP22.0-ER*, *AtHSP21*, and *AtHSP70T-2*) in WT and *MdHSFA1d*-overexpression lines at the normal temperature (22°C) and 1 h after exposure to the high-temperature (37°C) treatment.


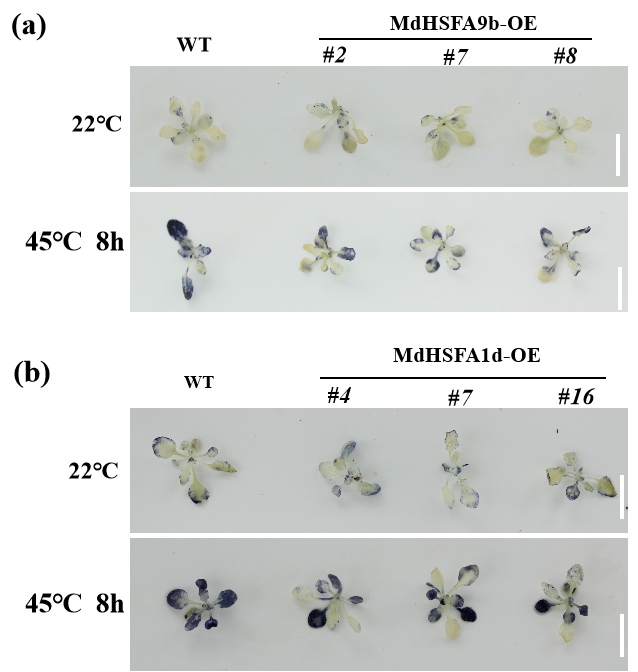


**Figure S5. Changes in the levels of accumulated ROS in Arabidopsis leaves under heat-stress conditions**.

(a) and (b) In situ accumulations of *MdHSFA9b* (a), *MdHSFA1d* (b), and superoxide radicals (O^2−^) before (upper panels) and after (lower panels) the heat treatment as revealed by nitro blue tetrazolium staining. Bar = 1 cm.


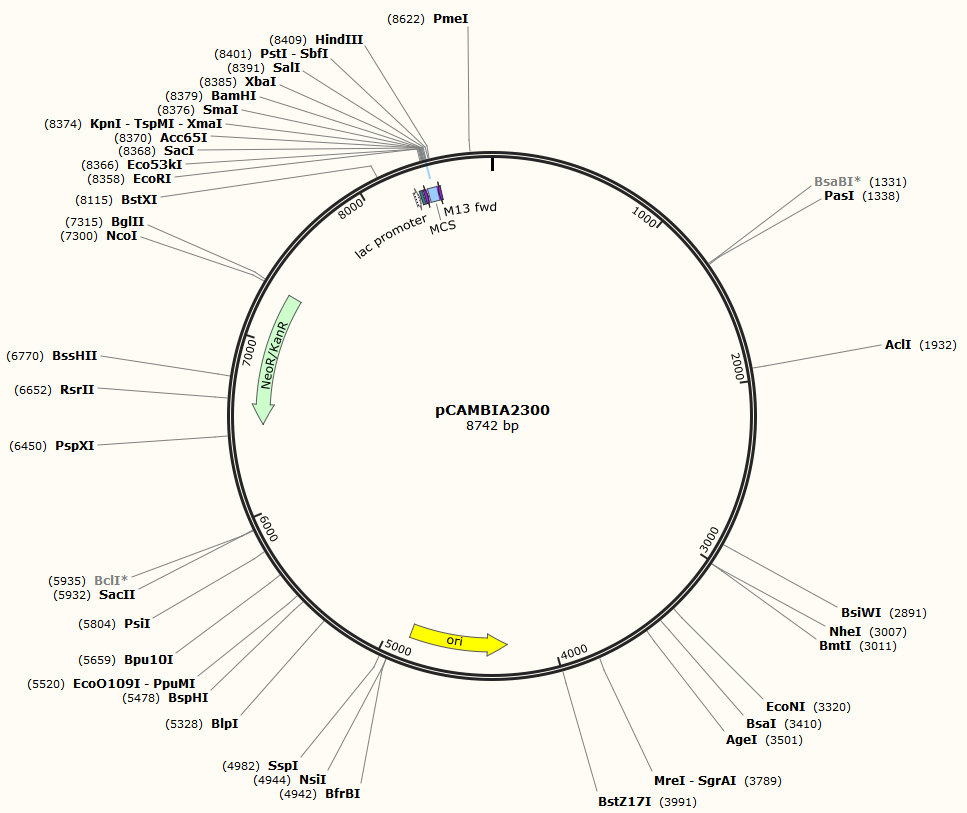

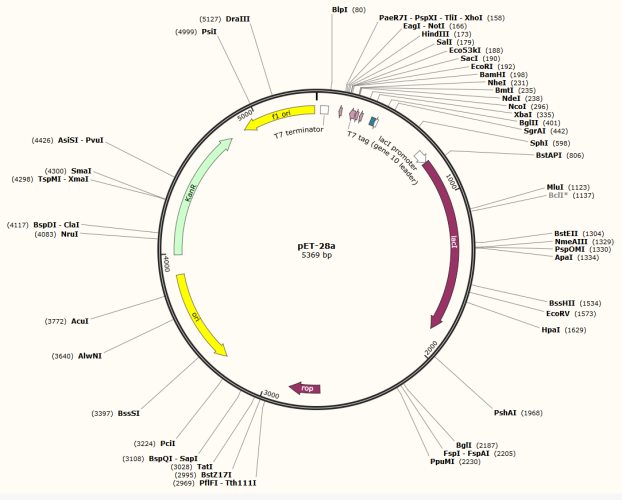

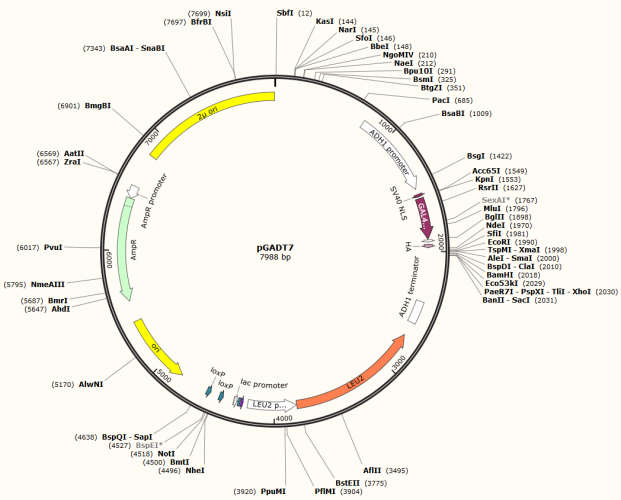

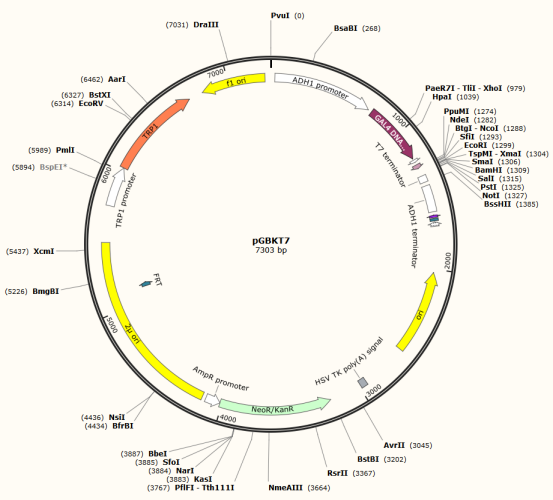

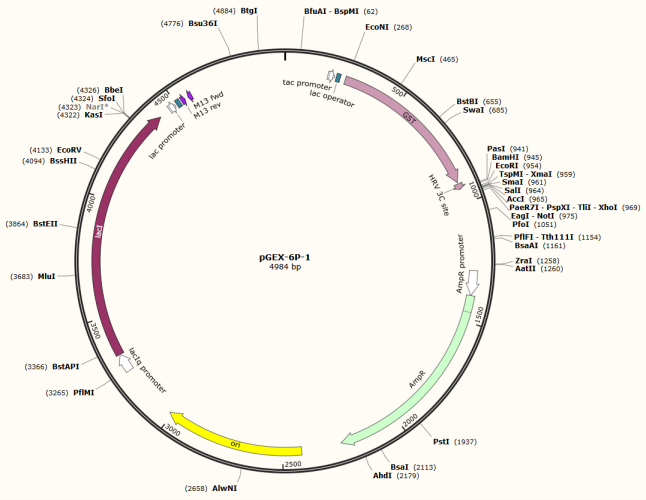

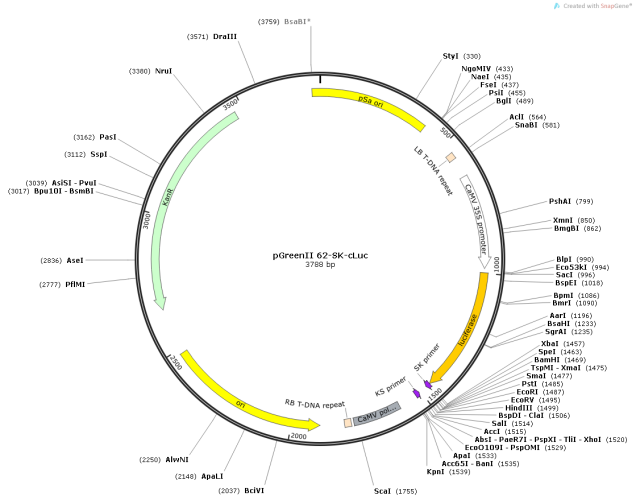

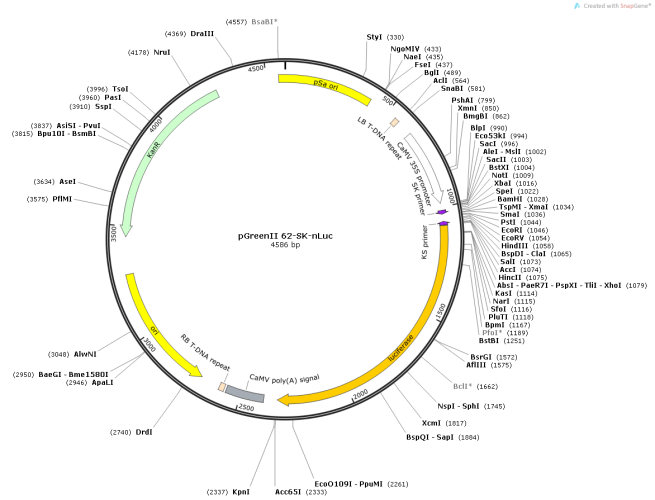

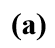

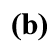

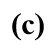

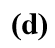

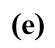

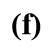

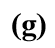


**Figure S6. Schematic diagram of vector.**

(a) pCAMBIA2300: using KpnⅠ and XbaⅠ for cloning of constructs. (b) pET28a: BamHⅠ and XhoⅠ. (c) pGEX-6P-1: BamHⅠ and EcoRⅠ. (d) pGBKT7: BamHⅠ and EcoRⅠ. (e) pGreenⅡ 62-SK-cluc: XbaⅠ and EcoRⅠ. (f) pGADT7: BamHⅠ and EcoRⅠ. (g) pGreenⅡ 62-SK-nluc: XbaⅠ and BamHⅠ.


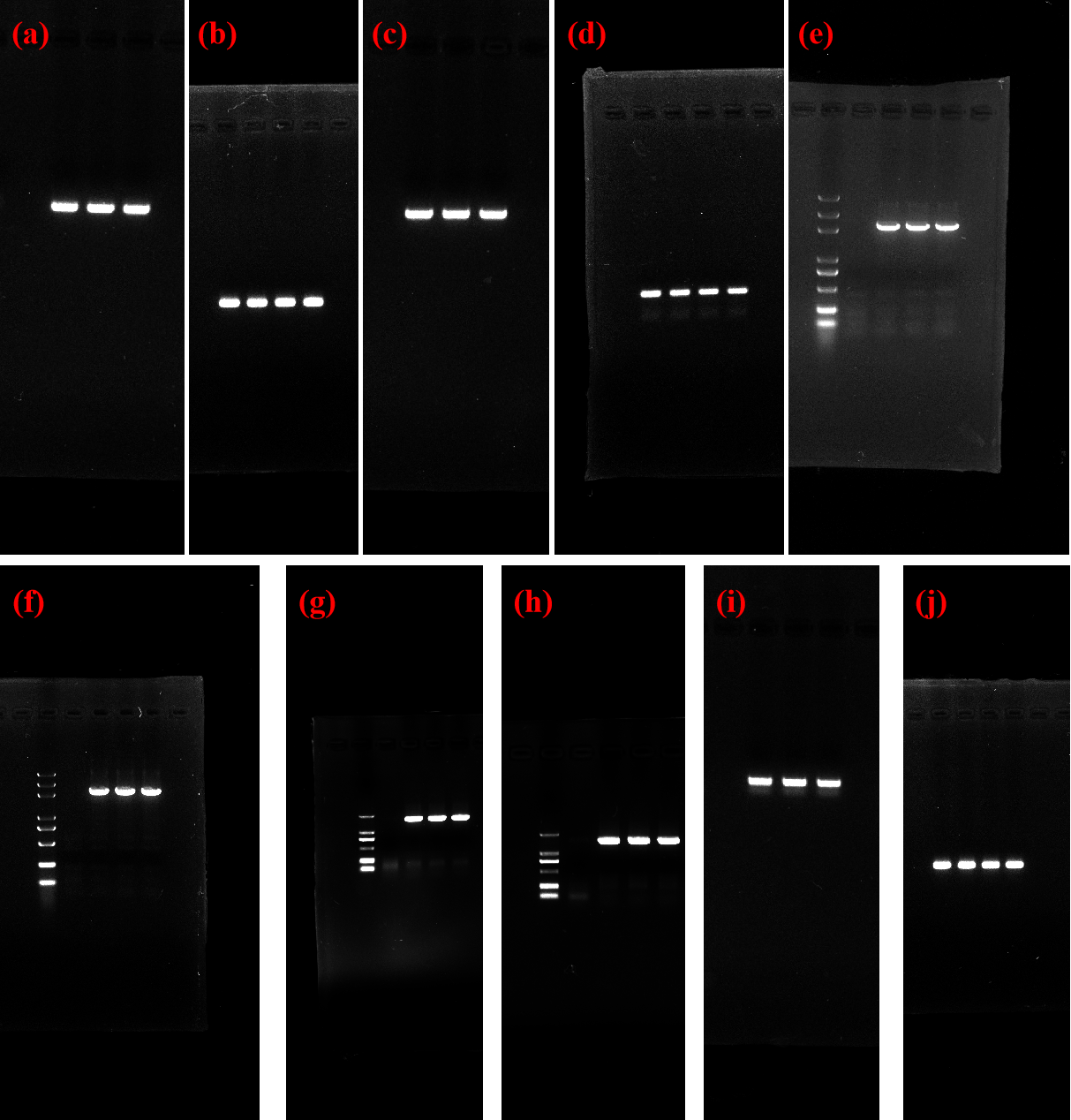


**Figure S7. Original image of nucleic acid electrophoresis.**

(a) Figure 3c: “*MdNup62*”. (b) Figure 3c: “*AtActin*”. (c) Figure 9c: “*MdHSFA9b*”. (d) Figure 9c: “*AtActin*”. (e) Figure 6d. (f) Figure S2a. (g) Figure S2b. (h) Figure S2c. (i) Figure S3c: “*MdHSFA1d*”. (j) Figure S3c: “*AtActin*”.


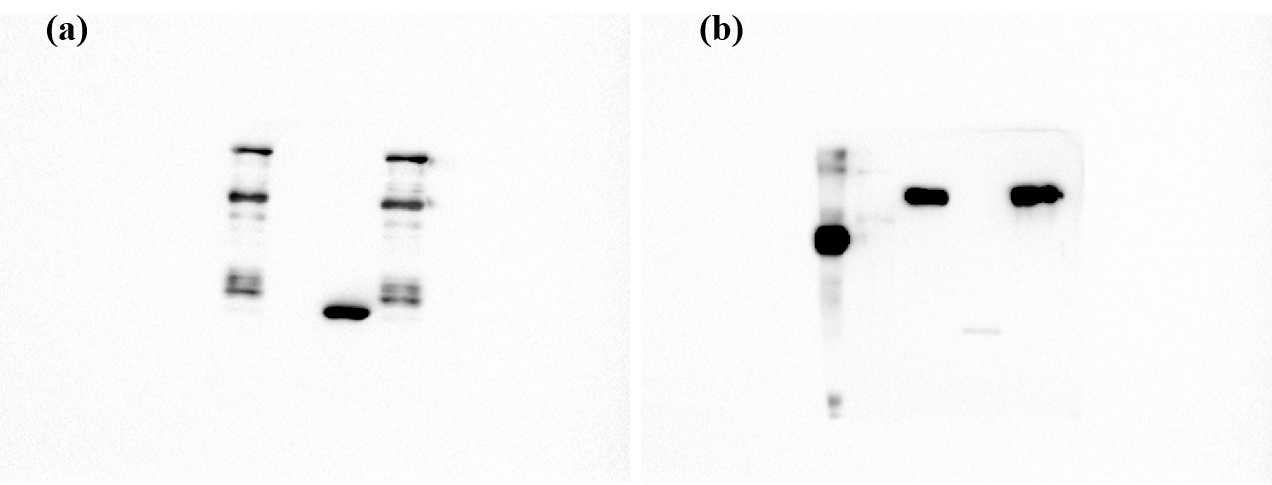


**Figure S8. Original image of Figure 7b.**

(a)Anti-GST. The channels from left to right are “GST-MdHSFA9b”, “His-MdNup62”, “GST+His-MdNup62”, and “GST-MdHSFA9b+His-MdNup62”. (b)Anti-His. The channels from left to right are “Marker”, “GST-MdHSFA9b”, “His-MdNup62”, “GST+His-MdNup62”, and “GST-MdHSFA9b+His-MdNup62”.
